# Nanoparticle–Cartilage Interaction: Pathology-Based Intra-articular Drug Delivery for Osteoarthritis Therapy

**DOI:** 10.1007/s40820-021-00670-y

**Published:** 2021-06-23

**Authors:** Xu Li, Bingyang Dai, Jiaxin Guo, Lizhen Zheng, Quanyi Guo, Jiang Peng, Jiankun Xu, Ling Qin

**Affiliations:** 1 Musculoskeletal Research Laboratory, Department of Orthopedics and Traumatology, The Chinese University of Hong Kong, Sha Tin, Hong Kong, SAR People’s Republic of China; 2 Joint Laboratory of Chinese Academic of Science and Hong Kong for Biomaterials, The Chinese University of Hong Kong, Sha Tin, Hong Kong, SAR People’s Republic of China; 3 Beijing Key Lab of Regenerative Medicine in Orthopedics, Key Laboratory of Musculoskeletal Trauma and War Injuries PLA, Institute of Orthopedics, Chinese PLA General Hospital, Beijing, People’s Republic of China; 4 Innovative Orthopedic Biomaterial and Drug Translational Research Laboratory, Li Ka Shing Institute of Health Sciences, The Chinese University of Hong Kong, Sha Tin, Hong Kong, SAR People’s Republic of China

**Keywords:** Nanoparticle, Drug delivery, Osteoarthritis, Articular cartilage, Nanomedicine

## Abstract

Osteoarthritis is the most prevalent chronic and debilitating joint disease, resulting in huge medical and socioeconomic burdens. Intra-articular administration of agents is clinically used for pain management. However, the effectiveness is inapparent caused by the rapid clearance of agents. To overcome this issue, nanoparticles as delivery systems hold considerable promise for local control of the pharmacokinetics of therapeutic agents. Given the therapeutic programs are inseparable from pathological progress of osteoarthritis, an ideal delivery system should allow the release of therapeutic agents upon specific features of disorders. In this review, we firstly introduce the pathological features of osteoarthritis and the design concept for accurate localization within cartilage for sustained drug release. Then, we review the interactions of nanoparticles with cartilage microenvironment and the rational design. Furthermore, we highlight advances in the therapeutic schemes according to the pathology signals. Finally, armed with an updated understanding of the pathological mechanisms, we place an emphasis on the development of “smart” bioresponsive and multiple modality nanoparticles on the near horizon to interact with the pathological signals. We anticipate that the exploration of nanoparticles by balancing the efficacy, safety, and complexity will lay down a solid foundation tangible for clinical translation.

## Highlights

- Nanoparticles hold considerable promise for controlling the local pharmacokinetics of therapeutic agents during osteoarthritis therapy.
- The advantages of nanoparticles, the bioactive design, the transports and interactions within cartilage, and therapeutic mechanisms are discussed.
- This review proposes future strategies to design more intelligent and multi-functional nanomaterials as delivery systems for cartilage therapy.

## Graphical abstract

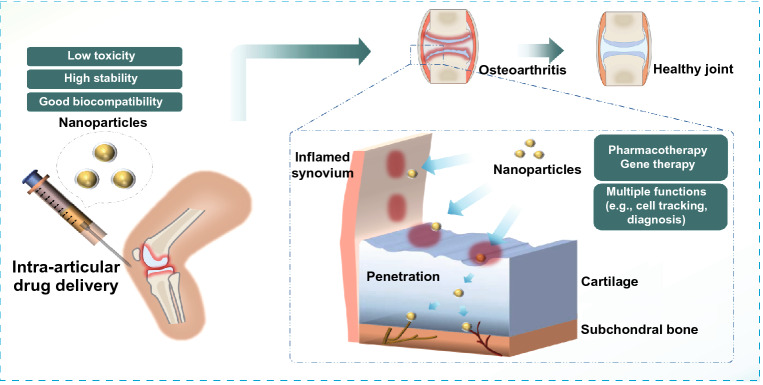

## Introduction

Osteoarthritis (OA) is the most prevalent chronic and debilitating joint disease and a leading cause of disability of elderly individual due to daily wear and tear of cartilage. Chronic pain, joint instability, stiffness, and radiographic joint space narrowing are major clinical symptoms, affecting about 10% of men and 18% of women over 60 years of age [1, 2]. Consequently, the resultant individual and socioeconomic burdens are huge [1, 2].

As aneural and avascular tissue, articular cartilage is of weak regeneration ability. Once damaged, it is hardly to be repaired and inescapable to degenerate. Because of the rapid clearance of synovial fluid and the barrier of dense natural cartilage extracellular matrix (ECM), the effectiveness of traditional intra-articular therapies, analgesics, glucocorticoids, and hyaluronic acid is still far from satisfactory [3]. To overcome these issues, nanoparticle is a desirable delivery system with the most suitable size to penetrate past the superficial zone of the cartilage and locally control the pharmacokinetics of therapeutic agents. As soon as nanoparticles are delivered into articular cavity, nano–cartilage interactions occur throughout their transport and penetration within the matrix. Apart from pain relief, nanoparticles-based therapy is also promising to attenuate cartilage degeneration and even promote regeneration. Armed with an updated understanding of the pathological features and OA pain, we may develop innovative nanoparticles for targeting multiple tissues, such as cartilage, nerves and/or blood vessels in synovium and subchondral bone, to enhance the therapeutic efficacy.

Herein, we review the pathological features of OA, limitations of current intra-articular therapies, and the advantages of nanoparticles for sustained drug delivery. Then, we summarize how to take advantages of these unique nanoscale properties, components, size, and surface chemistry, to facilitate their transports and interactions within cartilage. Furthermore, we highlight advances in the therapeutic mechanisms of nanoparticles. Finally, we place an emphasis on the design of the anticipated “smart” bioresponsive and multi-functional nanoparticles as the next-generation delivery system to interact with the pathological abnormalities and at the same time achieve controlled release. We anticipate that the exploration of nanoparticles by balancing the efficacy, safety, and complexity will lay down a foundation for clinical translations.

## Limitations of Current OA Therapy Demands Research and Development (R&D) of Effective Drug Delivery Systems

### Pathological Mechanisms of OA

The primary function of articular cartilage is to bear loading during motion. Articular cartilage is hyaline cartilage in nature with very limited support of blood vessels, nerves, or lymphatics. Articular cartilage mainly consists of highly specialized chondrocytes encapsulated in ECM. The slow turnover of ECM in cartilage makes the regeneration difficult in skeletally mature individuals. For example, it takes up to 25 years for the turnover of proteoglycans and the half-life of type II collagen is between 100 and 400 years [4–7].

Chondrocytes, which are sensitive to the changes of chemical and mechanical environment in OA, increase synthetic activity to generate collagen type X (matrix degradation associated products) at early stage of the disease [8]. Chondroptosis (apoptosis of chondrocytes) is increased in OA caused by oxidative and nitrosative stress, inflammatory cytokines, and mechanical stress [8]. At the same time, several inflammatory cytokines are produced, including interleukin (IL) 1β, IL 6, and tumor necrosis factor (TNF) α, and matrix-degrading enzymes (the matrix metalloproteinases (MMP) and a disintegrin and metalloproteinase with thrombospondin-like motifs (ADAMTS)) [8]. Ultimately, these enzymes mediate the degradation of cartilage ECM [8]. These catabolic factors also activate a series of pathways such as nuclear factor kappa B (NF-κB) and Wnt signaling, which play important roles during the pathological progress of OA [9–11]. At a later stage, inflammatory fluids filling the joint capsule cause swelling, more pain, and stiffness [8]. Cartilage becomes hypocellular with impaired metabolic flexibility [6].

**Fig. 1:**
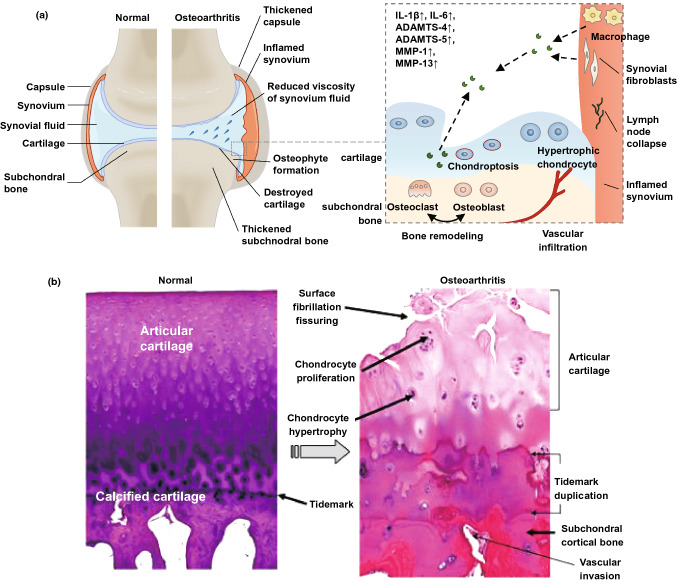
Pathological changes of OA. **a** Drawing of structural changes and signaling pathways of OA. **b** Histologic cross section of normal cartilage (left picture) and cartilage affected by end-stage OA (right picture). End-stage OA is characterized by articular cartilage injury, chondrocyte proliferation and hypertrophy, tidemark duplication, subchondral bone thickening, and vascular invasion. Reproduced with permission [277]. Copyright 2016, Elsevier Inc. Abbreviations: IL-1β, interleukin 1β; IL-6, interleukin 6; ADAMTS-4, a disintegrin and metalloproteinase with thrombospondin-like motifs 4; ADAMTS-6, a disintegrin and metalloproteinase with thrombospondin-like motifs 6; MMP-1, matrix metalloproteinases-1; MMP-13, matrix metalloproteinases-13; OA, osteoarthritis

There is an imbalance between catabolic and anabolic metabolism of articular cartilage in OA (Fig. 1) [12]. Although the synthesis of ECM increases, it is no longer able to fully compensate cartilage degradation [12]. MMP-13 is mainly in charge of degrading collagen and ADAMTS4 and 5 is for the destruction of aggrecan [13]. Decrease in the concentration of glycosaminoglycans (GAGs) and the disruption of the collagen orientation are therefore presented during OA. Consequently, the permeability of cartilage and interstitial fluid velocities within the matrix are increased [14]. Initially, changes of integrity at the surface disrupt cartilage composition and increase the susceptibility to physical forces [8, 15]. Without proper therapy, fissures will form in deep cartilage along with calcified cartilage zone expansion [16]. Additionally, the degradation of cartilage leads to the remodeling of subchondral bone and subchondral thickening [8, 15]. This process resembles chondrocyte differentiation during embryogenesis accompanied by the formation of osteophytes and subchondral cysts [8, 15]. Synovitis is another key feature of OA. Notable tissue hypertrophy, synoviocytes proliferation and increased vascularity contribute to the release of inflammatory factors [15]. Additionally, persistent joint inflammation leads to lymph node collapse and reduced lymphatic drainage, which contribute to severe synovitis and joint erosion [17].

### Inadequate Clinical Therapy Efficacy

Pain medication is the mainstay of pharmacotherapy for OA [18, 19]. Oral administration of nonsteroidal anti-inflammatory drugs (NSAIDs), cyclooxygenase-2 (COX-2) inhibitors, and acetaminophen has shown to soothe the pain and improve function [18–21]. However, significant adverse events and low local concentration of drugs are the main obstacles limiting their clinical applications. For example, oral administration of NSAIDs increases the risk of heart attack and induces kidney or gastrointestinal disorders [22].

Intra-articular drug delivery has various advantages over the systemic administration, such as increased local bioavailability throughout the joint capsule and fewer adverse events [3]. Intra-articular delivery of corticosteroids, hyaluronic acid, platelet-rich plasma and glucosamine or chondroitin can increase local bioavailability and serve as another strategy to reduce pain and relieve symptoms [3, 18–21, 23]. However, synovial fluid with complex biological composition and high viscosity affects drug’s properties and diffusion. The low residence time of drugs during articular cavity administration is mainly caused by the rapidly cleared from the synovial fluid [3]. The dense networks of collagen fibers and proteoglycans of cartilage ECM are also obstacles for drug absorption. Furthermore, cartilage ECM is avascular and densely packed with negative charged molecules, which particularly makes the diffusion of the negative charged drug even more difficult. Consequently, the half time of NSAIDs and soluble corticosteroids injected into the joint capsule is about 1–4 h, whereas hyaluronic acid can be cleared within 26 h [24, 25]. Repeated injections as the simplest way to increase therapeutic efficacy bring other problems such as increased risk of infection, joint disability, and the resultant high cost. Limitations of current pharmacological therapy for OA are concluded in Table 1. With the degeneration of cartilage, these therapies become less efficient, and the ultimate choice is joint replacement. Thus, drug delivery system of higher efficacy is required to overcome these obstacles.

**Table 1:** Current pharmacological therapy in OA and the limitations

Drug type	Action	Mechanism	Target tissue	Systemic or local treatment	Retention	Limitation
NSAIDs (e.g., ibuprofen; naproxen; celecoxib)	Pain relief	Anti-inflammation by COX enzymes inhibition	Inflammatory tissue in the articular cavity	Systemic	Not applicable	Low local concentration of drugs; almost no cartilage regeneration capacity. Adverse effect: risk of heart attack, stroke, kidney, liver, and stomach issues
				Local	1–4 h (half-life) [25, 276]	Provide short-term analgesic benefits; almost no cartilage regeneration capacity
Paracetamol	Pain relief	COX-2 inhibition	Inflammatory tissue in the articular cavity	Systemic	Not applicable	Low local concentration of drugs; almost no cartilage regeneration capacity Adverse effect: liver problems
Glucocorticoids	Pain relief	Anti-inflammatory actions by binding with intracellular glucocorticoid receptors	Inflammatory tissue in the articular cavity	Local	1–4 h (half-life) [25]	Provide short-term analgesic benefits; unclear balance between the benefits and potential harms; almost no cartilage regeneration capacity Adverse effect: allergic reaction especially after frequent injections (bleeding, and skin changes)
Hyaluronic acid	Viscosupplementation and pain relief	Lubrication and chondroprotection by binding with CD44 receptors	Synovial fluid, joint capsule, synovial membrane and cartilage	Local	26 h [24]	Improvement in symptoms or structure over placebo is unclear; low residence time; limited cartilage regeneration capacity Adverse effect: joint swelling or pain, and allergic reaction
Glucosamine	Nutraceuticals	Substrate for the biosynthesis of glycosaminoglycan chains	Cartilage and others	Systemic	Not applicable	Efficacy is controversial Adverse effects: mild upset stomach
Chondroitin sulfate	Nutraceuticals	Substrate for the biosynthesis of glycosaminoglycan chains	Cartilage and others	Systemic	Not applicable	Efficacy is controversial Adverse effects: mild upset stomach

COX-2, cyclooxygenase-2

### Advantages of Nanoparticles for the Treatment of Cartilage Disease

**Fig. 2:**
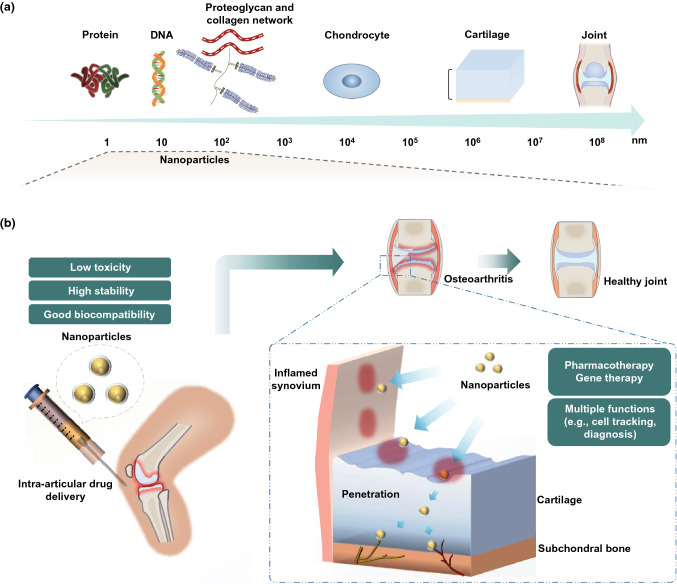
Properties and application schemas of nanoparticles for the treatment of cartilage disease. **a** Size of nanoparticles compared with different components in joint. **b** Application schemas of nanoparticles for intra-articular delivery

Nanoparticles refer to submicron particles with the dimension from 1 to 100 nm, which is about one thousand times smaller than chondrocytes (Fig. 2a). The controllable size endows nanoparticles the feasibility of direct intra-articular injections. Nanoparticles as carriers can incorporate drugs in the surface or matrix to protect drugs from enzymatic degradation, improve their penetrations across cartilage matrix, and modulate drug pharmacokinetics, which is beneficial for balancing the efficacy and the toxicity of therapeutic compounds (Fig. 2b). In order to optimize the degradation, toxicity, and mechanical properties, hybrid nanoparticles combining two or more components may have superior properties than single-component materials. By adjusting physicochemical properties or decorating with moieties, nanoparticles can be functionalized to target components and/or cells, e.g., chondrocytes, in the cartilage. Biocompatible and biodegradable materials such as polymers or solid lipids make up nanoparticles to enable controlled and sustained drug release. The increased specific surface area and surface to volume ratio are also beneficial for the dissolution and release of drugs [26]. Moreover, modifications can be performed by grafting other functional units for imaging. Other technological advantages includes high stability (e.g., long shelf life), feasibility of incorporation of both hydrophilic and hydrophobic substances and feasibility of variable routes of administration (including intra-articular injection or in combination with scaffold or hydrogel) [27].

Nanoparticle-based local gene transfer can alter the expression of the endogenous genes to prevent or slow the pathological progress of OA by introducing genes, such as DNA, mRNA, siRNA, and miRNA, into the target cells. Compared with the naked genetic molecules, nanoparticles hold potential to provide safe, efficient, and controllable gene delivery by manipulating properties such as encapsulation efficiency, stability, degradation, endocytosis, endosomal escape, and toxicity.

## Transports of Nanoparticles Within the Joint Cavity

### Pharmacokinetics and Biodistribution of Nanoparticles

**Fig. 3:**
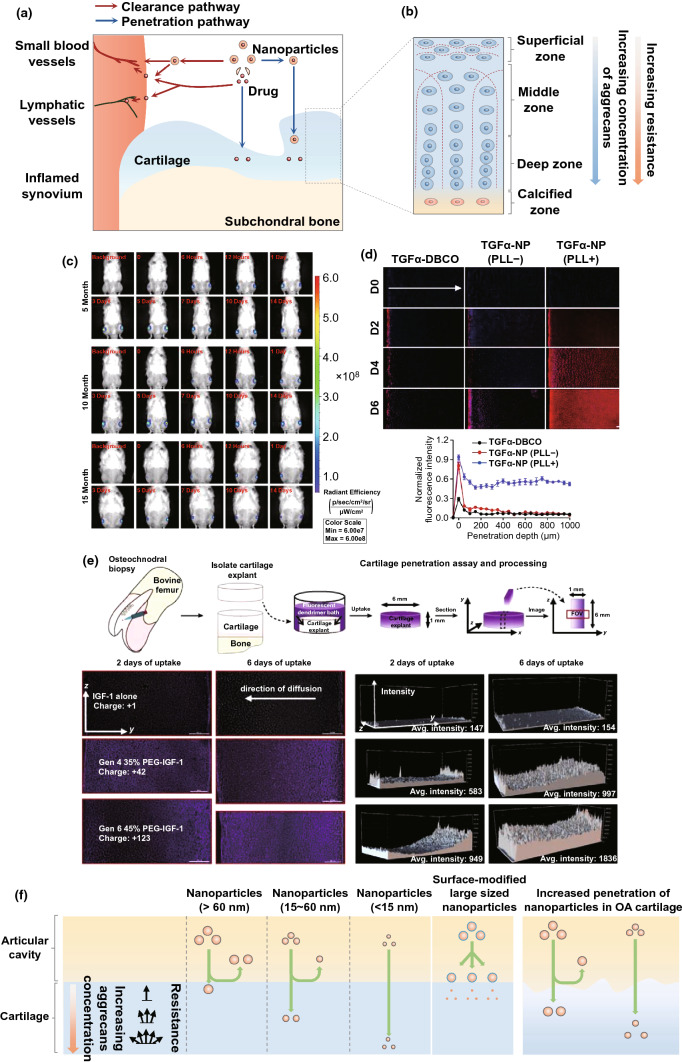
Interaction of nanoparticles with cartilage. **a** Clearance and biodistribution of nanoparticles within joint cavity. **b** Cartilage layers as barriers of drug penetration. **c** Retention of nanoparticles in OA and the contralateral joints in rats with different ages. Reproduced with permission [33]. Copyright © 2020 Elsevier B.V. **d** Penetration of 25.93 nm nanoparticles within bovine articular cartilage with similar joint cartilage thickness to human. Reproduced with permission [34]. Copyright © 2021 American Association for the Advancement of Science. **e** Penetration of 25.93 nm nanoparticles within bovine articular cartilage [40]. Reproduced with permission Copyright © 2018 American Association for the Advancement of Science. **f** Penetration of different sizes of nanoparticles into the cartilage matrix. Penetration depths of nanoparticles within cartilage matrix is in a size-dependent manner. Surface-modified nanoparticles with large size can be functionalized binding to the cartilage surface for drug release. Penetration of nanoparticles increases in OA cartilage compared with healthy cartilage Copyright © 2020 Elsevier B.V. **d** Penetration of 25.93 nm nanoparticles within bovine articular cartilage with similar joint cartilage thickness to human. Reproduced with permission [34]. Copyright © 2021 American Association for the Advancement of Science. **e** Penetration of 25.93 nm nanoparticles within bovine articular cartilage [40]. Reproduced with permission Copyright © 2018 American Association for the Advancement of Science. **f** Penetration of different sizes of nanoparticles into the cartilage matrix. Penetration depths of nanoparticles within cartilage matrix is in a size-dependent manner. Surface-modified nanoparticles with large size can be functionalized binding to the cartilage surface for drug release. Penetration of nanoparticles increases in OA cartilage compared with healthy cartilage

The proper space–time retention in the joint cavity is the prerequisite for ensuring nanoparticles' effective interaction with different components in the joint. Therefore, real-time monitoring of pharmacokinetics and biodistribution (PK/BD) of nanoparticles is important to define their therapeutic effect. Nanoparticle-based intra-articular delivery systems reduce the distribution to the reticuloendothelial organs and increase drugs’ half-lives by at least tenfold than free drugs [28, 29]. Pharmacokinetics of nanoparticles within cartilage depends on their chemical and physical properties, including size, charge, and surface chemistry, as well as the pathological state of joint cavity. Nanoparticles or their encapsulated drugs exit joints via the small blood vessels and lymphatic system (Fig. 3a) [30, 31]. The small blood vessels are the main channels for the clearance of small particles [30, 31]. In contrast, lymphatic pathway eliminates particles or their degradation products independently of their size [30, 31]. A study using mouse model proves that particles are preferentially drained through the iliac lymphatic nodes near the aortic bifurcation, and the remaining goes through hind leg lymphatic drainage to enter the inguinal lymphatic node [31].

Theoretically, the retention of drug carriers in cartilage depends on the net flux penetrating cartilage from synovial fluid and their efflux rate from the lymphatics and subsynovial capillaries. Therefore, the net influx penetrating cartilage from synovial fluid must reach therapeutic levels before the clearance to ensure effective drug delivery. If the concentration of nanoparticles within articular cavity reaches a high level immediately after the delivery. If the concentration of nanoparticles in synovial fluid is higher than that in the cartilage, the nanoparticles will penetrate into the cartilage matrix. When the concentration in synovial fluid decreases with the clearance of lymphatic and blood vessels, nanoparticles diffuse outward from cartilage into synovial fluid. Detailed pharmacokinetics of drug delivery systems has been well described in previous literature [32].

Synovial inflammation in OA increases capillary permeability and alters the lymphatic permeability (displayed initial compensatory expanding phase followed by a collapsed phase) [17]. Synovial fluid and serum tend to equilibrate through synovium intercellular gaps in a normal status; in OA, synovial inflammation-associated increased capillary permeability facilitates the elimination of nanoparticles from joint [31]. However, the clearance of nanoparticles in OA joint is impaired compared with the healthy joint, which may be associated with synovial thickening [33, 34]. Ageing associated metabolic changes, including less range of joint motion and decreased lubricating fluid, may also impede the clearance of nanoparticles. In a rat OA model, increased particle retention (in 14 days) has been found in 15-month rat knees, compared to 5- and 10-month rat knees (Fig. 3c) [33]. Although no animal study precisely recapitulates how the pathophysiology of OA affects the clearance of nanoparticles from lymphatic pathway, we can speculate that the initial 'expansion' phase of lymphatic vessels during moderate experimental arthritis may facilitate efficient lymphatic clearance; in contrast, the collapsed phase of lymphatic vessel characterized by lymphatic vessel structural damage and loss of contraction may reduce lymphatic clearance of nanoparticle or their encapsulated drugs [17].

### Size-Dependent Penetration Within Cartilage Matrix

The dense ECM and avascularity structure determine the transports and interactions of nanoparticles within cartilage. Human knee articular cartilage is about 2 to 4 mm thick, which mainly consists of three zones—the superficial (tangential) zone (~ 10% to 20%), the middle (transitional) zone (~ 40% to 60%), and the deep zone (~ 30 to 40%) (Fig. 3b) [32, 35]. Superficial zone contains a high number of flattened chondrocytes and collagen fibers aligned tightly parallel to the articular surface. The mesh size of collagen type II fibrillar network in the superficial zone is about 50–60 nm [36, 37]. The space between the side chains of the proteoglycan network is about 20 nm [37, 38]. This zone with the good tensile properties is responsible for resisting the sheer, tensile, and compressive forces. The middle (transitional) zone is responsible for the resistance to compressive forces that contains proteoglycans and thicker obliquely organized collagen fibrils [32, 35]. The spherical chondrocytes are distributed at low density [32, 35]. In deep zone, chondrocytes are parallel to the collagen fibers and columnar to the joint line [32, 35]. Proteoglycan content is the highest, and the resistance to compressive forces is the greatest [32, 35]. The calcified zone containing scarce and hypertrophic chondrocytes distinguishes the deep zone from the subchondral bone [32, 35].

The depth at which the nanoparticles can diffuse depends on both the pores in the cartilage nano/microstructure and the size of the particles. The increased network density of proteoglycan with the thickness of cartilage makes the penetration of nanoparticles more difficult. Whereas larger sized nanoparticles do not penetrate into cartilage, smaller ones are capable of penetrating deeper (Fig. 3f). As the pore size of collagen type II fibrillar network is about 50–60 nm in the superficial zone [36, 37], nanoparticles beyond this size may not be able to enter the cartilage matrix efficiently. Human-thickness bovine cartilage is commonly employed to investigate the penetration of nanoparticles. The accumulation of 38-nm nanoparticles of poly(propylene sulfide) (PPS) in bovine cartilage cartilage matrix is 14.9-fold higher than 96 nm nanoparticles after intra-articular delivery for 24 h, although most of the nanoparticles are still withheld in the cartilage surface [37]. Poly[L-lysine-block-poly(ε-caprolactone)] (PLL)–polycaprolactone (PCL) nanoparticles (25.93 nm) can efficiently bound to the surface of bovine cartilage explants with articular cartilage thickness similar to that of human at day 2 and gradually penetrated inside by at least 1 mm by day 6 (Fig. 3d) [34]. Given the space between the side chains of the proteoglycan network is about 20 nm, small (≤ 15 nm) nanoparticles can easily enter the cartilage matrix by binding and penetrating anionic cartilage tissue [37, 38]. For example, a study proves that 15-nm micelles are better than 138-nm-diameter liposomes in penetrating bovine articular cartilage [39]. At 4-h post-nanoparticles treatment, micelles have already penetrated across the superficial and middle zones of bovine articular cartilage [39]. Similarly, amine terminal polyamidoamine (PAMAM) dendrimers functionalized with variable molar ratios of poly(ethylene glycol) (PEG) (diameter < 10 nm) exhibit full penetration of human-thickness bovine cartilage with a 70% absorption rate (Fig. 3e) [40]. Such nanoparticles as a drug delivery system increase the residence time of insulin-like growth factor 1 (IGF-1) by tenfold for up to 30 days [40]. Another study also proves that nanoparticles diameters ~ 5 nm are capable of penetrating throughout the full thickness (1 mm) of the normal bovine cartilage within 24 h, while an obvious penetration gradient is also found with the treatment of particles diameters ~ 10 nm [41]. Even so, this does not mean that the smaller the diameter of the nanoparticles, the better therapy efficacy. Small-size nanoparticles may be easily cleared from the joint via sub-synovial capillaries and lymphatics more rapidly. In contrast, larger-sized nanoparticles with the advantage to deliver a more sustained payload are not necessarily unsuitable for drug delivery. Therefore, there are plenty of rooms for sorting out the optimal size of nanoparticles to maximize the efficacy. Alternatively, large-sized nanoparticles floating in the articular cavity need to release sufficient drug to enable the drug’s penetration within cartilage. If nanoparticles can be functionalized binding to the cartilage surface by modifying surface-functional properties, their released drugs may be able to diffuse to deeper layer of cartilage by minimizing the clearance effects (Fig. 3f).

Although rodent models are widely used to investigate the OA treatment and its underlying mechanism, large animal with thicker cartilage is more suitable for exploring the transport kinetics of nanoparticles. Therefore, bovine cartilage is the most widely used for studying the penetration of nanoparticles in the past [39–41]. The thickness of cartilage negatively affects the effective diffusion of nanoparticles [32]. Giving that the thickness of cartilage increases with animal size, nanoparticles are more likely to be cleared in large animal’s cartilage [14, 32, 42, 43]. Similarly, the outward diffusion of nanoparticles is also proportional to the square of the cartilage thickness [32]. Once the concentration of nanoparticles reaches therapeutic levels, the theoretical retention time also increases with cartilage thickness [14, 32, 42, 43]. Of note, the decrease in proteoglycan and collagen in OA usually increases the pore size and affects the diffusion of drugs (Fig. 3f) [14]. Nanoparticles can penetrate deeper into the proteoglycan-depleted cartilage than normal cartilage [41]. Large molecules exhibit higher diffusivities benefiting the most from the increased pore size [14].

### Targeting Therapy to Facilitate Nanoparticle–Cartilage Interaction

**Fig. 4:**
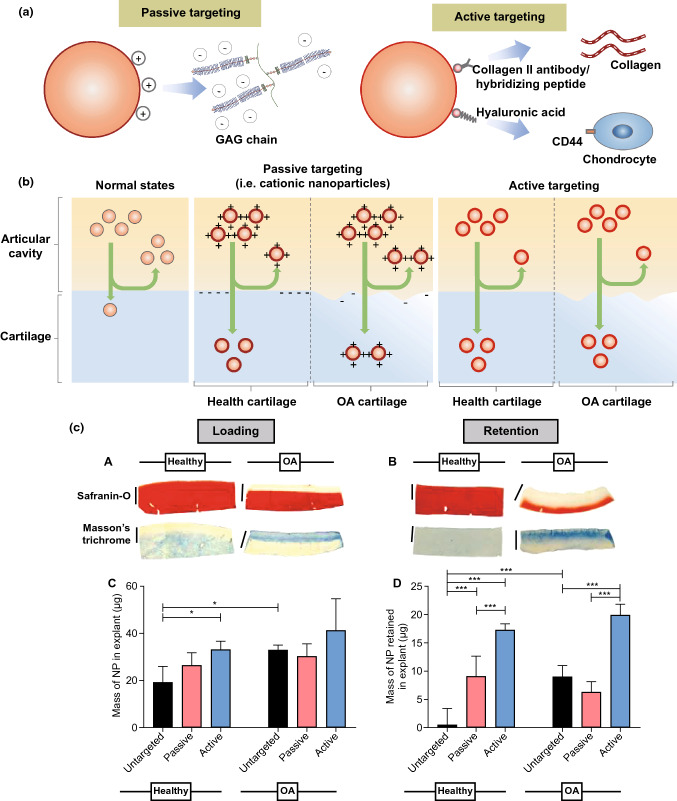
Nanoparticle design for cartilage targeting therapy. **a** Strategies for passive and active targeting. **b** Effects of passive and active targeting on the penetrations of nanoparticles within healthy and OA cartilage. **c** Interactions of passive and active targeting nanoparticles with healthy and OA-mimicked cartilage. Reproduced with permission [54]. Copyright 2019, Acta Materialia Inc. Published by Elsevier Ltd.

Desirable nanoparticles should be able to functionally target to specific components and/or cells of the cartilage. The strategies can be divided into passive targeting and active targeting (Fig. 4a, b and Table 2). Passive targeting is established by improving the physicochemical properties such as size, charge, surface chemistry for preventing unspecific interactions, which needs to fully consider the unique characteristics of cartilage ECM. Negative charged cartilage offers the unique opportunity to utilize electrostatic interactions with the positive charged nanoparticles to achieve passive targeting. Accelerated augment transport, uptake and intra-tissue binding of the positive charged nanoparticles shorten the time of intra-cartilage drug to reach therapeutic concentration and extend the half-life in vivo (Fig. 4a). For example, cationic globular proteins and dendrimers can target to the anionic cartilage matrix via electrostatic attraction [34, 40, 44]. With both approaches, electrostatic interactions between positively charged nanoparticles and the negative fixed charge of cartilage ECM can be optimized to augment the transport, uptake and intra-tissue binding of such nanoparticles. For example, cationic surfactants such as didodecyldimethylammonium bromide (DMAB) can help nanoparticles achieve passive targeting to improve their retentions in cartilage [45]. The retention of DMAB PLGA nanoparticles is fourfold higher than the corresponding negatively charged nanoparticles with the presence of synovial fluid [45]. Another approach is to use the cationic domain of a therapeutic drug, such as cytokines, to enable the binding to cartilage. For example, FGF family with a cationic heparin-binding domain binds heparan sulfate GAG chain in cartilage [46, 47]. The positively charged amino acids in the heparin-binding (HB) domain can bind to the negatively charged sulfate and carboxyl groups in heparin. In addition, heparin-binding domains can also be used to attach to other cytokines such as IGF-1 to accelerate the transport inside cartilage. For example, HB-IGF-1 fabricated by binding the heparin-binding domain of HB-EGF to the amino-terminus of IGF-1 maintains the transduction of IGF signaling through the IGF receptor and displays prolonged therapeutic effect in OA model [48–50]. Besides the physicochemical properties of nanoparticles, synovial fluid and the disease state of the cartilage also affect their retention in cartilage. For example, the retention of cationic DMAB PLGA nanoparticles decreases by 50% in the presence of synovial fluid compared with saline [45]. The possible reason is that hyaluronic acid as an anionic, non-sulfated glycosaminoglycan in synovial fluid may influence the passive targeting of positively charged nanoparticles. More importantly, the disease state of OA negatively affects passive targeting therefore compromises the ability of the positively charged nanoparticles to penetrate the matrix. For example, the retention of cationic DMAB PLGA nanoparticles displays a threefold reduction in OA cartilage compared with healthy cartilage [45].

**Table 2:** The summary of nanoparticle-based systems for targeting cartilage microenvironment

Category of target therapy	Targeted tissue	Targeting ligand	Carrier	Size	Species and model	Targeting efficiency	References
Passive target	Anionic cartilage	Cationic macromolecules-amine terminal polyamidoamine (PAMAM) dendrimers	Dendrimers	< 15 nm	Rat surgical OA model/ bovine ex vivo	Enhanced residence time in rat knees by tenfold for up to 30 days	[40]
	Anionic cartilage		55 mol% PEG-PCL)/20 mol% PLL-PCL/25 mol% DSPE-PEG5K-N3	25.93 nm	Mice OA model/ bovine ex vivo	Fluorescence signal in joints injected with nanoparticles was much higher than those injected with free drug	[34]
	Anionic cartilage	Eudragit RL100	Cationic polymer (VivoTag®)	Between 100 and 150 nm	Rat model	70% of the nanoparticles were retained in the joint for 1 week	[44]
	Anionic cartilage	Quaternary ammonium cation	PLGA	260–290 nm	Bovine ex vivo	Cationic nanoparticles demonstrated sixfold greater retention than anionic nanoparticles in healthy cartilage,	[45]
	Anionic cartilage	Poly(allylamine) hydrochloride (PAA)	PLGA	172.4 ± 20.5 nm	Mice OA model	Significantly improved nanoparticle associations with both healthy and OA-like cartilage	[54]
Active target	Collagen II α1	Collagen II targeting peptide (WRYGRL)	PPS	38 nm and 96 nm	Bovine ex vivo	Targeted articular cartilage up to 72-fold	[37]
	Collagen II	Type II collagen antibody	Immunoliposomes	250 nm	Dunkin-Hartley guinea pig OA model	Bind to damaged but not normal cartilage	[51]
	Collagen II α1	Collagen II targeting peptide (WRYGRL)	Ferritin	About 22 nm	Mice OA model	–	[53]
	Collagen	Collagen type II binding peptide	PLGA	180.2 ± 8.0 nm	Mice OA model	Cartilage accumulation was increased in OA relative to healthy knees	[54]
	Chondrocytes	Hyaluronic acid	Polylactide	700 nm	in vitro model	Captured more by articular cells (chondrocytes)	[57]
	Chondrocytes	Hyaluronic acid	Bovine serum albumin	150.5 ± 8.4 nm	Sprague Dawley rats	Exhibited improved uptake by chondrocytes through a receptor-mediated active uptake mechanism	[59]
	Activated macrophages	Folic acid	Silver	400 nm	Mice model of collagen-induced arthritis	Passively accumulate into inflamed joints	[191]

DSPE-PEG5K-N3, 1,2-distearoyl-sn-glycero-3-phosphoethanolamine-N-[azido(polyethylene glycol)-5000]; OA, osteoarthritis; PEG-PCL, poly (ethylene glycol)-polycaprolactone; (PLL-PCL), poly[L-lysine-block-poly(ε-caprolactone)]; PLGA, poly(lactic-co-glycolic acid; PPS, poly(propylene sulfide)

Active targeting is established by using conjugation chemistries to attach affinity ligands to the surface of the nanoparticles (Fig. 4a, b). Targeted cell can recognize decorated nanoparticles through ligand–receptor interactions. For example, nanoparticles decorated with collagen type II antibodies can specifically bind to cartilage and facilitate drug release inside the cells [40, 51]. In addition, several types of peptides termed the collagen hybridizing peptide/ collagen-targeting peptide have been developed as moiety of nanoparticles to specifically bind to denatured collagen strands by re-forming a triple-helical structure in a fashion [52–54]. ECM (including collagen and proteoglycan) surrounding the chondrocytes has higher turnover rate [55, 56]. Chondrocytes targeting therapy can be a suitable strategy to assemble collagen and proteoglycan distribution. CD44 is expressed by chondrocytes which can be used for active targeting [57–59]. Nanoparticles covered with hyaluronic acid can specifically binds to CD44, provoking the internalization [57–59].

The extent of cartilage accumulation and joint biodistribution for the two types of targeting-system is differently affected by disease states [54]. Accumulation of active nanoparticles is increased in OA cartilage compared with healthy cartilage, indicating that active targeting strategies may be advantageous for drug delivery to diseased cartilage (Fig. 4c) [54]. However, from a translational aspect, passive targeting strategies requires fewer modifications, making production easier and more controllable, therefore reducing the cost and facilitating the translational.

### Interactions with Targeted Cells

**Fig. 5:**
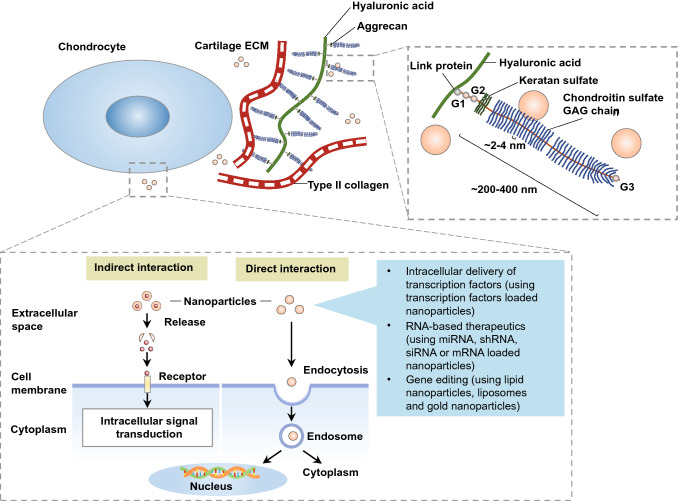
Possible interactions of nanoparticles with targeted cells

Penetrations of nanoparticles within cartilage result in either direct contact through cell uptake, or indirect interaction through release of nanoparticle-containing materials with targeted cells (Fig. 5). For the indirect interaction, therapeutic agents can be released to the cartilage matrix and affect cellular communications via receptor ligand interactions. For the direct interaction, nanoparticles may enter the targeted cells by endocytosis-based uptake pathways. Nanoparticles are typically confined within intracellular vesicles, such as endosomes, phagosomes, or macropinosomes [60]. Endocytosis-based uptake pathways can be further categorized into phagocytosis, macropinocytosis, clathrin‐ and caveolae‐mediated endocytosis, and clathrin‐ and caveolae‐independent routes, which are regulated and mediated by specific type of lipids and transport proteins [60, 61]. Most nanoparticles accomplish intracellular delivery by endocytosis and endosomal escape; in particular, intracellular gene delivery for in situ cellular reprogramming can be closely associated with endocytosis. Since nucleic-acid biomolecules are negatively charged, the penetration into cartilage ECM and diffuse across negatively charged phospholipid cell membranes become extremely difficult; as such, nanoparticles are developed to overcome the obstacles. The major existing approaches based on the platform of nanoparticles include intercellular delivery of transcription factors, RNA-based therapeutics and gene editing [62]. After being delivered in the cytoplasm, genes will directly regulate mRNA levels or translocated to the nuclei. Elucidate fundamental mechanisms of how nanoparticles gain access into chondrocytes are still critical for the mediation of physicochemical parameters, including size, charge, shape, and surface modifications) to increase therapeutic efficacy. Interactions of nanoparticles with targeted cells are also possibly affected by the severity of disease. Increased activities of catabolic enzymes in OA may negatively influence the indirect interaction with targeted cells by changing both properties of nanoparticles and physiological activities of the targeted cells. In addition, uptake pathways of nanoparticles in normal chondrocytes and diseased chondrocytes, including hypertrophic chondrocytes and, apoptotic chondrocytes, may be different and the mechanisms behind remain to be investigated.

### Summary of Size Design

The optimal size of the nanoparticles should be designed according to the target sites for treatment. If the target sites are inflamed synovial fluid or synovial membrane rather than deep layer of cartilage, large sized, non-penetrating drug carriers can be used to avoid the clearance of blood vessels and lymphatics. If the target sites are superficial zones of cartilage, the size of nanoparticles should be at least smaller than the pore of collagen type II fibrillar network (50–60 nm) [36, 37]. After binding to the cartilage surface, nanoparticles can release the encapsulated drugs at deeper sites. If the target sites are full thickness of cartilage, the size of nanoparticles should be even smaller than the pore of proteoglycan network (20 nm) [37, 38]. In addition, active or passive targeting strategies can be used to reduce articular cavity clearance and increase the retention time within cartilage matrix.

## Materials Design of Nanoparticles

**Fig. 6:**
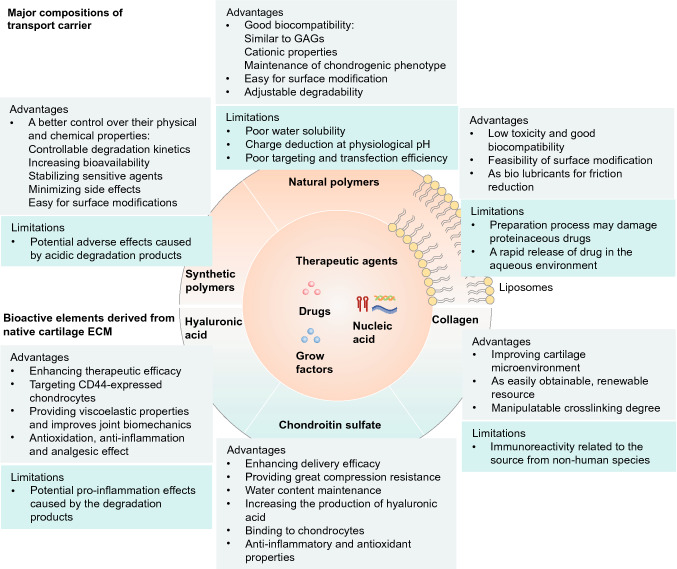
Compositions and properties of biomimetic nanoparticles for the treatment of cartilage disease

Nanoparticles as delivery systems mainly contain transport carriers, bioactive elements and therapeutic agents (e.g., drugs and genes) (Fig. 6). Therapeutic agents are encapsulated by transport carriers, which are mainly responsible for controlling the pharmacokinetics of therapeutic agents including ensuring efficient concentration within cartilage while decreasing undesirable side effects. Bioactive elements are engineered for locally enhancing delivery efficiency and improving the cartilage microenvironment. Compositions of nanoparticles and their focused pathological pathways are summarized in Table 3.

**Table 3:** Compositions of nanoparticles and their focused pathological pathways within joint cavity

Carrier		Agents	Pathological pathway	References
Materials	Category			
Solid lipid nanoparticles	Synthetic polymer	Celecoxib	Anti-inflammation	[28]
PLGA nanoparticles	Synthetic polymer	Betamethasone sodium phosphate	Anti-inflammation	[29]
Polymeric micellar nanoparticles	Synthetic polymer	TGFα	EGFR signaling (protect against cartilage degeneration and attenuate pain)	[34]
PEG-PAMAM dendrimers	Synthetic polymer	IGF-1	Promote chondrocyte survival, proliferation, and biosynthesis of cartilage matrix macromolecules; anti-inflammatory	[40]
Liposome	Liposome	–	–	[39]
Liposome	Liposome	Anti-collagen type II monoclonal antibodies	Targeting cartilage (mainly collagen)	[51]
MMP-13/pH-responsive ferritin nanocages	Protein	Hydroxychloroquine	Anti-synovial and cartilage inflammation	[53]
PAA-PLGA nanoparticles	Synthetic polymer	Collagen type II binding peptides	Targeting cartilage (mainly collagen)	[54]
PLA	Synthetic polymer	Not applicable	Targeting CD44-positive chondrocytes	[57]
Hyaluronic acid	Natural polymer			
Bovine serum albumin	Protein	Brucine	Stimulate chondrocyte proliferation and inhibit the early apoptosis	[59]
Hyaluronic acid	Natural polymer			
Folic acid-modified silver nanoparticles	Metal	Not applicable	Targeting M1 macrophages	[191]
PLGA	Synthetic polymer	TGF-β1; bFGF	Stimulate chondrocyte proliferation and differentiation	[70]
PLGA	Synthetic polymer	Anti-TNF-α siRNA	TNF-α secreted Inflammatory cells	[63]
PLGA	Synthetic polymer	Anti-COX2 siRNA	Anti-inflammation	[64]
PLGA	Synthetic polymer	SOX9 pDNA	Chondrogenesis induction	[71]
Chitosan nanoparticle	Natural polymer	Berberine chloride	Promotes chondrocytes survival and matrix production	[82]
Folic acid-, DEAE-, and PEG-modified chitosan nanoparticle	Include natural and synthetic polymer	TNF-α siRNA	Anti-TNF-α secreted Inflammatory cells	[83]
Hyaluronic acid	Natural polymer	TGF-β1 plasmid DNA	Chondrogenesis induction	[84]
Chitosan	Natural polymer			
Thiolated glycol chitosan nanoparticles	Include natural and synthetic polymer	TNF-α siRNA	Anti-TNF-α secreted Inflammatory cells	[85]
Hyaluronic acid	Natural polymer	Cytokine response modifier A	Targeting synovial inflammation via inhibition of IL-1β generation	[87]
Chitosan	Natural polymer			
1,2-distearoyl-sn-glycero-3-phosphocholine liposome	Liposome	D-glucosamine sulfate	Anti-inflammatory effect and lubrication	[89]
Liposome	Liposome	Celecoxib	Anti-inflammation	[92]
Liposome	Liposome	Dexamethasone or diclofenac	Anti-inflammation	[93]
Hyaluronic acid	Natural polymer			
Collagen	Natural polymer			
Hyaluronic acid	Natural polymer	Plasmid	Chondrocyte gene delivery	[101]
Chitosan	Natural polymer			
Solid lipid	Solid lipid	Prednisolone	Anti-inflammation	[102]
Hyaluronic acid	Natural polymer			
Polyethylenimine	Synthetic polymer	Plasmid DNA	Chondrocyte and synoviocyte gene delivery	[103]
Chitosan	Natural polymer			
Hyaluronic acid-conjugated thermosensitive polymer	Include natural and synthetic polymer	Not applicable	Reduce pro-inflammatory cytokines and preserve epiphysis thickness	[107]
Solid lipid nanoparticles	Solid lipid	Diacerein	Anti-inflammation	[122]
Chondroitin sulfate	Natural polymer			
Platinum nanoparticles	Metal	Not applicable	Not applicable	[123]
Chondroitin sulfate	Natural polymer			
Decellularized extracellular matrix	Natural materials	Not applicable	Produce physical and biochemical supportive cues for chondrogenesis	[130]
Poly(3‐sulfopropyl methacrylate potassium salt)‐grafted mesoporous silica nanoparticle	Synthetic polymer and inorganic compound	Diclofenac sodium	Anti-inflammatory effect and lubrication	[140]
Dopamine melanin nanoparticle	Natural compound	Not applicable	Anti-inflammatory and chondro-protective effects by inhibiting intracellular ROS	[145]
Peptide nanoparticle	Peptide	NF-κB siRNA	Attenuate early inflammation by enhancing AMPK signaling while suppressing mTORC1 and Wnt/β-catenin activity	[146]
Perfluorooctylbromide nanoparticle	Organic compounds	Fumagillin	Targeting angiogenic blood vessels	[148]
Block copolymer nanoparticle	Copolymer	IL-1Ra protein	Inhibit IL-1-mediated signaling	[150]
Hyaluronic acid	Natural polymer	Tocilizumab	Inhibit IL-6-mediated signaling	[151]
Gold	Metal			
Solid lipid nanoparticles	Solid lipid	Chloroquine	Inhibit TNF-α-mediated signaling	[152]
Poly(N-isopropylacrylamide) nanoparticle	Synthetic polymer	Anti-inflammatory peptide KAFAKLAARLYRKALARQLGVAA (KAFAK)	Inhibit IL-6 and TNF-α-mediated signaling	[153]
Poly nanoparticle	Synthetic polymer	Anti-inflammatory peptide KAFAK	Inhibit IL-6 and TNF-α-mediated signaling	[154]
Poly(N-isopropylacrylamide)	Synthetic polymer	Anti-inflammatory MK2-inhibiting cell-penetrating peptide KAFAK	Suppress pro-inflammatory IL-6 expression	[155]
Thiolated glycol chitosan	Natural polymer	Notch1 siRNA	Anti-inflammatory effect targeting Notch1 signaling	[157]
Manganese dioxide nanoparticles	Metallic compound	Not applicable	Targeting ROS pathway	[161]
Silica nanoparticles	Inorganic compound	IGF-1	Promote chondrogenesis	[171]
PLGA nanoparticles	Synthetic polymer	KGN	Promote chondrogenesis	[131]
Chitosan	Natural polymer	KGN	Promote chondrogenesis and diagnose	[186]
Fe_3_O_4_	Metallic compound			
Silk fibroin	Natural materials	KGN	Promote chondrogenesis	[132]
PLGA nanoparticle	Synthetic polymer	pDC316-BMP4-EGFP plasmid	Promote chondrogenesis	[133]
Folic acid-conjugated hyaluronic acid nanoparticle	Natural polymer	Not applicable	Target activated macrophages for diagnosis	[190]
Fe3O4	Metallic compound	Not applicable	Promote chondrogenesis	[227]
Poly(NIPAm-co-AMPS-AAc-BAC) nanoparticle	Synthetic polymer	Anti-inflammatory MK2-inhibiting peptide	Anti-inflammation	[237]
Gold	Metal	FITC-modified-a disintegrin and metalloproteinase with thrombospondin motif-4-specific peptide	Diagnosis of degrading aggrecan	[248]
Donor–acceptor-type-conjugated polymer	Synthetic polymer	Tocilizumab	Inhibit IL-6-mediated signaling	[252]
PLGA nanoparticle	Synthetic polymer	Not applicable	Neutralize proinflammatory cytokines, and provide chondroprotection	[197]
Neutrophil membrane	Cell membrane			
Macrophage-derived microvesicle	Cell membrane	Tacrolimus	Mimic macrophages	[195]
PLGA nanoparticle	Synthetic polymer			

AMPK, adenosine monophosphate-activated protein kinase; bFGF, basic fibroblast growth factor; BMP4, bone morphogenetic protein 4; COX2, cyclooxygenase-2; DEAE, diethylethylamine; EGFP, enhanced green fluorescent protein; EGFR, epidermal growth factor receptor; FITC, fluorescein isothiocyanate; IGF-1, insulin-like growth factor 1; IL-1, interleukin-1; IL-1Ra, interleukin-1 receptor antagonist; IL-6, interleukin-6; KAFAK, KAFAKLAARLYRKALARQLGVAA; KGN, kartogenin; MK2, mitogen-activated protein kinase-activated protein kinase 2; MMP-13, matrix metallopeptidase 13; mTORC1, mammalian target of rapamycin complex 1; NF-κB, nuclear factor kappa-light-chain-enhancer of activated B cells; PAA, poly(allylamine) hydrochloride; pDNA, plasmid DNA; PEG, poly(ethylene glycol); PAMAM, amine terminal polyamidoamine; PLGA, poly(lactic-co-glycolic acid; ROS, reactive oxidative species; siRNA, small interfering RNA; SOX9, SRY-related HMG-box 9; TGFα, tumor necrosis factor alpha; TGF-β1, transforming growth factor-beta 1; TNF-α, tumor necrosis factor α

### Major Compositions of Transport Carriers

#### Synthetic Polymers

Biodegradable synthetic polymers such as PCL, poly (glycolic acid) (PGA), D, L-poly (lactic acid) (D, L-PLA), poly (l-lactic Acid) (PLLA), and their copolymer polylactic-co-glycolic acid (PLGA) are frequently used biodegradable polymers. Because of the promising mechanical characteristics, high biocompatibility, and versatility of chemistry, some of them (e.g., PLA, PGA and PLGA) have been approved by the US Food and Drug Administration (FDA) and European Medicines Agency (EMA) as carriers for drug delivery in humans. Biodegradable synthetic polymers as nanocarriers for target delivery can increase bioavailability, protecting instable agents (e.g., proteins and genes), and minimizing toxicity effects. In addition, the production cost of synthetic polymer material is often lower than that of natural polymer material while the shelf time is longer.

PLA and PLGA have been widely used as drug delivery systems in animal studies of OA therapy [29, 57, 63, 64]. The major advantage of synthetic polymers is a good control over their physical and chemical properties. The surface properties can be tailored for specific biomedical applications. Because the negative charge on synthetic polymers (e.g., PLGA) surface may reduce the ability to enter the negative charged cartilage matrix, cationic surface modifications of PLGA-based nanoparticles by using cetyltrimethylammonium bromide, polyethyleneimine, poly(2-dimethylamino)ethyl methacrylate, didodecyldimethylammonium bromide, and chitosan is necessary for improving drug delivery efficacy [65–67]. Additionally, it is feasible to incorporate both hydrophilic and hydrophobic substances in synthetic polymers [68]. For example, surface modification of (hydrophobic) PLA and PLGA with hydrophilic PEG to form an amphiphilic block copolymer facilitates a high drug loading and efficient delivery within tissue [68]. Hydrophobic PLGA core can be used for encapsulation of drugs and genes, while the hydrophilic PEG shell prevents the surface from aggregation, opsonization, and phagocytosis and prolongs systemic circulation time [68, 69]. Moreover, the degradation speed can be manipulated to control the release of therapeutic agents. The hydrolytic degradation of PLGA in vivo depends on hydrolysis of the polymers to generate the lactic and glycolic acid monomeric components which can be tailored through controlling polymer molecular weight, copolymerization, and functionalization. By adjusting the size and structure, degradation kinetics of synthetic polymer nanoparticles can be controlled to achieve dosage‐ and site‐specific drug delivery [63, 64, 70, 71]. However, the potential drawback is that their acidic degradation products, including caproic acid, succinic acid, valeric acid, and butyric acid as degradation product of PCL, may aggravate cartilage inflammation and matrix degradation.

#### Natural Polymers and Their Derivatives

Polysaccharides including chitosan, dextran, alginate, and cellulose derivatives are the most versatile natural polymers that broadly used in drug delivery. Among them, biologically active natural GAG analogues such as chitin and chitosan show therapeutic potential for inter-articular drug delivery. Their physical and chemical resemblance of cartilage ECM determines their major advantages—low toxicity and good biocompatibility. As the most abundant polysaccharide in the marine ecosystem and second in nature (after cellulose), chitin can maintain the morphology of chondrocytes and preserve the synthesis of ECM [72, 73]. Chitosan (poly-β-1,4-linked glucosamine) as derivative conversed from alkaline N-deacetylation of chitin has been widely studied for the delivery of therapeutic agents to cartilage [74]. The molecular structure of chitosan is similar to GAGs in normal cartilage, determining its good biocompatibility for maintaining the chondrogenic phenotype and proliferation activity [75, 76]. The cationic property of chitosan makes it different from most neutral or negatively charged polysaccharides. The positive charge allows it easily to bind to the negatively charged cartilage ECM or form electrostatic complexes with other negatively charged polymers. Because of the mild processing conditions and chemical reactivity, chitosan has been widely used in the field of surface modification [77]. The existence of β-(1,4) glycosidic bonds between d-glucosamine and N-acetyl-d-glucosamine provides possibilities to be modified for altering properties such as solubility, adhesion and stability [78]. Adjustable degradability is another property of chitosan determined by the deacetylation degree [79]. Lowly deacetylated chitosan degrades fast [79]. Besides, chitosan possesses non-toxicity, hydrophilicity, anti-inflammation, anti-bacterial and anti-fungal properties, and wound-healing effects [78].

Chitosan nanoparticles have been widely used as stable delivery systems for either controlled drug release or as a non-viral gene vector for transferring genes [80, 81]. For drug delivery, intra-articular injection of drug-loaded chitosan nanoparticles decreases the concentration of therapeutic agents in plasma, increases the retention time in synovial fluid, and therefore effectively ameliorates OA [82]. For gene delivery, the combination of chitosan nanoparticles with DNA or siRNA is able to transfect the chondrocytes [83–87]. The small size makes it more easily to be taken up by endocytosis of chondrocyte [26]. However, before further application, a series of problems need to be resolved such as poor water solubility, charge deduction at physiological pH, and poor targeting and transfection efficiency [88].

#### Liposomes

Liposomes have been investigated as micro- or nanocarriers to change pharmacokinetics and biodistribution of drugs in the treatment of OA. Besides the low toxicity and good biocompatibility, liposomes can incorporate both hydrophilic and hydrophobic molecules and exist feasibility of surface modification to present targeting option and prolong the retention in cartilage. Phospholipids act as effective bio-lubricants for friction reduction and maintenance of mobility of synovial joints. Therefore, it is possible to achieve both sustained drug delivery and improved lubrication by using liposomes at the same time [89]. Liposomes of larger size display good retention in joint cavity and therefore are better for the improvement of joint boundary lubrication [90, 91]. Although the size (above 100 nm) of liposomes determines the poor penetration within cartilage, sustained drug release within articular cavity can be provided through liposome dissolution. With a high encapsulation efficiency (as high as 90%), anti-inflammatory drug-loaded liposomes displays more promising outcome than the therapeutic entities they contain in pain control and cartilage protection [92, 93]. Nevertheless, the preparation procedure contains the mix with organic solvents which may damage proteinaceous drugs. Additionally, the aqueous environment of the synovial fluid may lead to a rapid burst release of drug.

### Components Derived from Native ECM

The compositions of natural ECM provide templates for the selection of bioactive and biomimetic materials. Mainly produced by chondrocytes, ECM is composed of collagens (60–85% of dry weight), proteoglycans (15–40% of dry weight), and other non-collagenous proteins, and responsible for retaining water and maintaining mechanical properties that are anisotropic, nonlinear, inhomogeneous and viscoelastic (Fig. 4) [94]. Type II collagen is the principal collagen (90% to 95% of collagen) in ECM, and the fibers are intertwined with proteoglycan aggregates [94, 95]. Proteoglycan aggregates are high molecular weight molecules which are composed of GAGs covalently bound to a central protein [94, 95]. Type II collagen and proteoglycans are mainly responsible for the tensile and compression strength, respectively [94, 95]. GAGs as high molecular weight linear polysaccharides can be divided into four classes including hyaluronic acid, keratan sulfate, dermatan sulfate, and chondroitin sulfate [94, 95]. Aggrecan is the main proteoglycan that its core protein contains three globular domains and two glycosaminoglycan-attachment domains [95]. An N-terminal globular domain of aggrecan interact with hyaluronic acid to form proteoglycan aggregates [95]. The chondroitin sulfate chains attach to the chondroitin sulfate domain, which is responsible for the high fixed charged density and the ability to resist compressive loads in cartilage [95]. Chondrocytes receive nutrients depending on the diffusion of synovial fluid and also indirectly interact with components of synovial fluid including hyaluronic acid, lubricin, glucose, aggrecan, chondroitin sulfate, keratan sulfate, and water [96].

#### Hyaluronic Acid

As a non-sulfated glycosaminoglycan, hyaluronic acid maintains a constant concentration in cartilage and synovial fluid as a space filler [97]. In synovial fluid, hyaluronic acid functions in lubrication, hydration balance, matrix structure, and steric interactions to provide viscoelastic properties [97]. The binding to ECM molecules and cell surface receptors makes hyaluronic acid as a modulator of cellular behaviors including differentiation, proliferation, development, and recognition [98]. When OA occurs, the decreased average molecular weight and concentration of hyaluronic acid aggravates damage to the cartilage [99].

Balazs and Denlinger proposed the concept of viscosupplementation for the treatment of OA [100]. The intra-articular injection of hyaluronic acid can recover the rheological properties of synovial fluid which further promote the synthesis of endogenous hyaluronic acid and consequently improve joint biomechanics [100]. Hyaluronic acid also exerts pharmacologic actions including antioxidation, anti-inflammation, analgesic effect and chondroprotection [97]. Hyaluronic acid has emerged as the moiety of the drug and gene carriers for OA treatment [86, 87, 101, 102]. Multiple functional units in hyaluronic acid enable it to be chemically modified by other moieties which is beneficial for enhancing therapeutic efficacy or decreasing toxicity. For gene delivery, the chondrocyte transfection efficiency of nanoparticles with hyaluronic acid is higher than that without hyaluronic acid [101, 103]. CD44, as a receptor of hyaluronic acid, is also highly expressed by synovial lymphocytes, macrophages, and fibroblasts in inflamed joints [104–106]. Hyaluronic acid-based nanoparticles can carry anti-inflammatory drugs targeting these cells as an active targeting strategy. Hyaluronic acid-decorated nanoparticles can also target CD44-expressed chondrocytes and therefore lead to better targeting in cartilage [58]. Besides improving chondrocytes targeting efficiency, hyaluronic acid-based nanoparticles persist longer retention than free drugs and those without hyaluronic acid [102]. More interestingly, hyaluronic acid with the conjugation of a thermosensitive polymer displays spontaneous formation of nanoparticles after intra-articular injections to a murine OA model. Those nanoparticles offer a prolonged residence time (exceed 21 days near the injection site) to reduce pro-inflammatory cytokines and preserve epiphysis thickness [107]. Nevertheless, it should be noted that larger hyaluronic acid molecules are depolymerized producing low molecular weight hyaluronic acid, leading to excess inflammatory response [108].

#### Chondroitin Sulfate

As highly sulfated and linear polysaccharide, chondroitin sulfate makes up the main constituent of GAGs, accounting for 20% weight/dry weight of adult articular cartilage [109–113]. Chondroitin sulfate is composed of a chain of alternating sugars (N-acetylgalactosamine and glucuronic acid) and has an important role in maintaining the structural integrity of cartilage [110, 111]. The turnover of chondroitin sulfate affects the mechanical property of ECM and modulates the homeostasis of chondrocytes. Disaccharide unit heterogeneity and sulfates on disaccharide units determine the negative charge of chondroitin sulfate polymer and its biological activities in cartilage such as the maintenance of the water content and the great resistance to compression [114]. Chondroitin sulfate increases the production of hyaluronic acid by synoviocytes to maintain viscosity [115]. The capacity to bind chondrocyte is 5- to 7-times higher than hyaluronic acid and keratin sulfate [116]. In addition, chondroitin sulfate inhibits the synthesis and activities of proteolytic enzymes, nitric oxide, and other substances and thus prevent cartilage matrix from damage [117]. Chondroitin sulfate can reduce the nuclear translocation of NF-κB to inhibit inflammation, favor the synthesis of hyaluronic acid and collagen II, and therefore limit matrix degradation [117, 118]. In OA, the degradation of chondroitin sulfate in cartilage is increased, which further increases water content in cartilage ECM to induce a hypertrophy-like morphology of chondrocytes and MMP-13/ADAMTS5 production [119, 120]. The European League Against Rheumatism (EULAR) gave chondroitin sulfate the highest recommendation for the treatment of OA [121]. Although the oral drug delivery has been commercialized, there is still challenge in securing instability of delivery system to achieve its efficacy. As the moiety of nanoparticles, chondroitin sulfate displays the potential to increase delivery efficiency in joints while without leading to toxicity [122, 123]. The hydrophilicity property of chondroitin sulfate-based nanoparticles increases water solubility of hydrophobic drugs, prolongs articular cavity retention, and promotes cartilage targeting [122, 123].

#### Collagen and Acellular ECM

As the most abundant biopolymer in the human, collagen becomes an easily obtainable, renewable resource for the recovery of cartilage ECM structure and function. Besides the capacity to resemble the cartilage microenvironment, collagen exhibits an extremely high biocompatibility with low immunogenicity and is biodegradable and bioresorbable. Type II collagen can minimize chondrogenic hypertrophy, prevent joint destruction and pain for the treatment of OA [124, 125]. Moreover, the degree of cross-linking can be manipulated and the physical properties such as size, surface area, and absorption capacity, are easy to configure, which makes collagen-based nanoparticles a prime candidate for controlling drug release. Commercial native collagen products have been extracted from chicken sternum [124]. One potential drawback, however, is the immunoreactivity related to the source from non-human species.

Acellular ECM which theoretically contains all the bioactive compositions is the nature’s template to provide adequate nutrient support for tissue repair [126, 127]. Therefore, acellular ECM is biodegradable and do not elicit adverse immune responses. The properties to induce chondrogenic differentiation and promote cartilage regeneration have been proved [128, 129]. As the major component of nanoparticles, it is capable of supporting viability and proliferation of chondrocyte [130].

### Intra-articular Delivery Choices

At present, most of the basic research focuses on the direct intra-articular injection of nanoparticles to solve the clinical problem of rapid drug clearance. Direct intra-articular injection of nanoparticles can minimize systemic exposure and increase local bioavailability by providing controlled and sustained drug release. However, clinical intra-articular drug injection is used in most cases to treat mild to moderate OA. Attempts to use nanoparticles for the treatment of severe OA are more desirable. If nanoparticles can be accumulated more at the severely defective sites, the therapeutic outcome could be better than that evenly distributed in the articular cavity. The combination of hydrogels or scaffolds with nanoparticles can enhance the stability of nanoparticles and extend the retention of drugs following intra-articular injection [131–133] (Table 4). In addition, scaffolds or hydrogels can affect cell survival and provide matrix for cell homing and regeneration [131–136].

**Table 4:** Combination of nanoparticles with scaffold/hydrogel for intra-articular delivery

Scaffold/hydrogel		Nanoparticle		Conclusion	References
Materials	Function	Materials	Function		
Silk fibroin and PEGDMA hydrogel	A semi‐degradable system for providing a stable scaffold for chondrogenesis	TGF-β1- and bFGF-loaded PLGA nanoparticles	For controlled dual delivery within the construct for improved proliferation and facilitated chondrogenic differentiation	Allow for dosage- and site-specific multiple growth factor delivery	[70]
Chitosan scaffold	Three-dimensional carrier for the nanoparticles	Hyaluronic acid/chitosan/TGF-β1 pDNA nanoparticles	Drug delivery for chondrogenesis	Enhance in vitro cartilage tissue engineering	[84]
Si-HPMC hydrogel	For cartilage repair	Laponite	Nanoreinforcement	Construct an interpenetrating network which enhances the hydrogel mechanical properties	[137]
Collagen-based scaffolds	As a scaffold for chondrogenesis	IGF-1-loaded silica nanoparticles	Promote chondrogenesis	Improve therapeutic intervention for the targeted and controlled treatment of articular cartilage lesions	[171]
Hyaluronic acid hydrogel	Enhance the stability of nanoparticles, alleviate burst release, and provide matrix for cell homing and regeneration	KGN-encapsulated PLGA nanoparticle	Promote chondrogenesis	Make the chondrogenesis efficient and persistent	[131]
Silk fibroin scaffold	Modulate nanoparticles release, and provide matrix for cell homing and regeneration	KGN-encapsulated silk fibroin nanoparticle	Promote chondrogenesis	Sequential release of pro-migratory and pro-chondrogenic molecules to induce endogenous chondrogenesis	[132]
PLLGA scaffold	Modulate nanoparticles release, and provide matrix for cell homing and regeneration	pDC316-BMP4-EGFP plasmid-loaded PLGA nanoparticle	Promote chondrogenesis	Improve in vivo chondrogenesis	[133]

bFGF, basic fibroblast growth factor; BMP4, bone morphogenetic protein 4; EGFP, enhanced green fluorescent protein; IGF-1, insulin-like growth factor 1; KGN, kartogenin; PEGDMA, poly(ethylene glycol) dimethacrylate; pDNA, plasmid-DNA; PLLGA, poly(L-lactic-co-glycolic acid); Si-HPMC, silated hydroxypropylmethyl cellulose; TGF-β1, transforming growth factor-beta 1

Hydrogels consisting cross-linked hydrophilic polymers for retaining water can mimic three-dimensional structure of the ECM and thus improve lubrication. With good biocompatibility and high permeability for nutrients, hydrogels can fill cartilage defects of any size in a minimally invasive way. Various hydrogel systems containing nanoparticles have been reported in the literature for articular cartilage repair [70, 137]. However, low mechanical strength is a major shortcoming of hydrogels. Since loading patterns affect the diffusion process of therapeutic agents [138], therapy based on nanoparticles alone is difficult to provide enough mechanical support at the region of severe wear and tear. The combination with scaffold is a strategy to solve the problem especially for the repair of large defect in weight-bearing area. An ideal biodegradable scaffold should favor cell survival and alleviates the further wear and degradation of the cartilage to support the biomechanical environment, which in turn provides a proper microenvironment for the controlled release of nanoparticles. In addition, nanoparticles can also provide support for the stability of hydrogels and scaffolds. For example, laponite nanoparticles can construct an interpenetrating network which enhances the hydrogel mechanical properties [137]. Further, the combination of nanoparticles with scaffolds or hydrogels is possible to achieve dosage- and site-specific multiple drug delivery [70].

### Summary of Material Design According to the Pathology Features

Since each material has its own characteristics, the adequate combination of different materials can improve the efficiency of drug delivery while circumvent individual shortcomings. The material selection needs to be considered for its own characteristics and the difference in target sites during the pathological processes. For example, liposomes with larger size are more suitable for articular cavity drug delivery and viscosupplementation for prevention of OA. In the next section, we discuss the therapeutic mechanism of nanoparticles during the pathological progress of OA.

## Therapeutic Schemes According to the Pathology Mechanisms

### Prophylactic Administration

#### Viscosupplementation

**Fig. 7:**
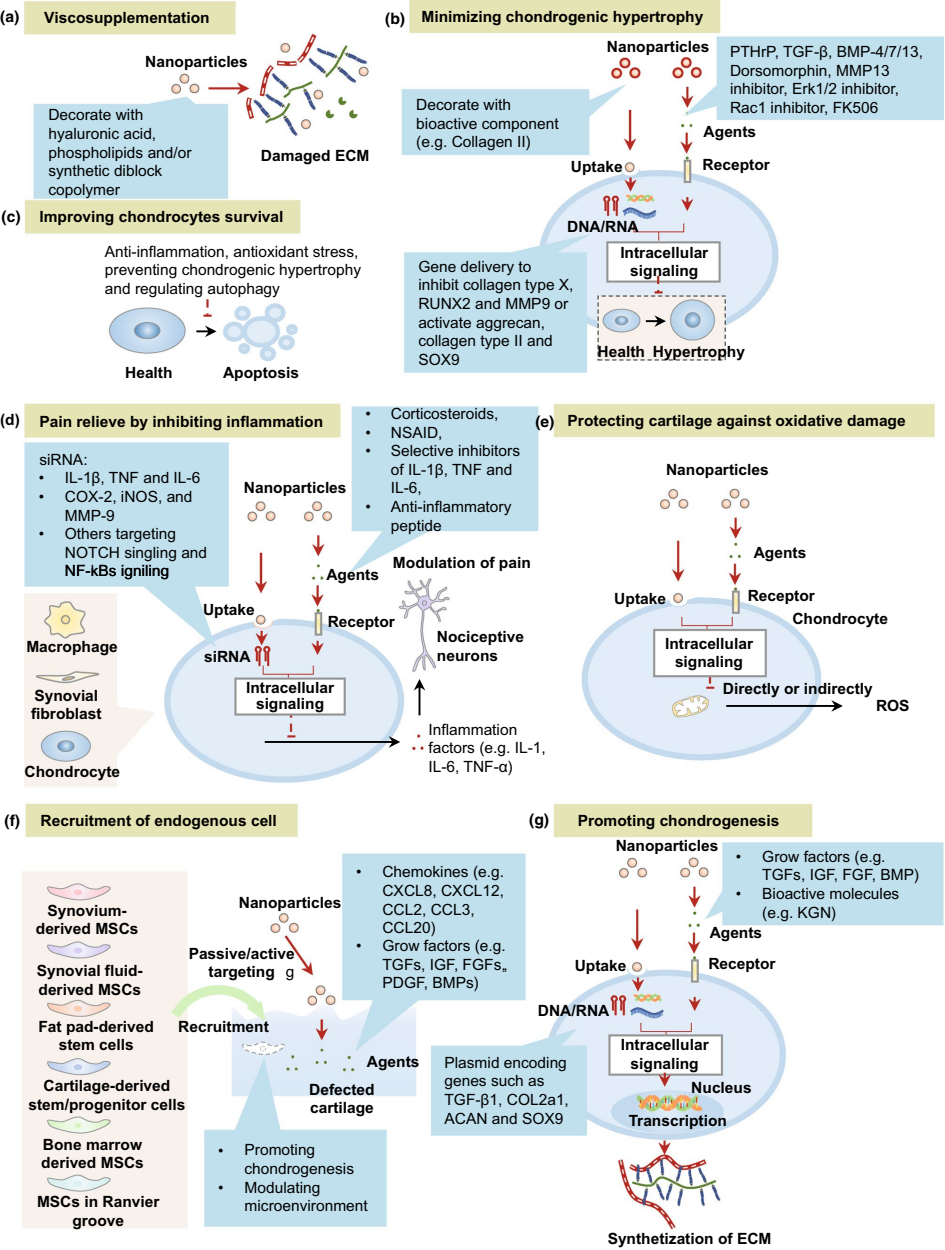
Uptake pathways and therapeutic mechanisms of nanoparticles in OA. The major mechanisms include **a** lubrication improvement, **b** chondrogenic hypertrophy prevention, **c** cell survival regulation, **d** pain relief by inflammation inhibition, **e** anti-oxidative damage, **f** recruitment of endogenous stem cells, and **g** chondrogenesis promotion. Abbreviations: ACAN, aggrecan; BMP 4/7/13, bone morphogenetic proteins 4/7/13; CCL 2/3/20, C–C motif chemokine ligand 2/3/20; COL2a1, collagen type II alpha 1 chain; COX 2, Cyclooxygenase-2; CXCL 8/12, chemokine (C-X-C motif) ligand 8/12; Erk1/2, extracellular signal‑regulated protein kinase 1/2; FGF, fibroblast growth factors; FK506, tacrolimus; IGF, Insulin-like growth factor; IL 1β/6, Interleukin 1β/6; iNOS, inducible nitric oxide synthase; KGN, kartogenin; MMP 9, matrix metalloproteinases; NF-κB, nuclear factor kappa-B; NSAID, nonsteroidal anti-inflammatory drugs; PDGF, Platelet-derived growth factor; PTHrP, parathyroid hormone-related protein; Rac1, Ras-related C3 botulinum toxin substrate 1; ROS, reactive oxygen species; SOX 9, SRY-Box transcription factor; TGFs, transforming growth factors; TNF, tumor necrosis factor

OA progress is associated with lubrication deficiency caused by the age-related degradation of glycoprotein (i.e., hyaluronic acid) [96, 139]. Nanoparticles combining lubrication protect against OA (Fig. 7a). As mentioned above, nanoparticles with components such as hyaluronic acid and phospholipids all are conductive to lubrication improvement and the maintenance synovial joint mobility. Synthetic diblock copolymer to mimic the functional domains of lubricin is also possible to be components of nanoparticles for lubrication improvement [139]. Polyelectrolyte polymer brushes can reduce friction coefficient via the hydration lubrication mechanism [140]. The combination with nanoparticle represents an effective approach to enhanced lubrication capability [140].

#### Cartilage Maintenance by Minimizing Chondrogenic Hypertrophy

Hypertrophic chondrocytes with a significant increase in cell size and volume degrade their surroundings and therefore accelerate the progression of OA [141]. The high expression of osteogenic differentiation-related genes appears to be associated with production of mineralized ECM proteins and calcification of articular cartilage [141]. Prevention of hypertrophy is a potential therapeutic strategy to facilitate the retardation of osteophytes and slow down OA progression (Fig. 7b).

High expressions of collagen type X, runt-related transcription factor 2 (RUNX2), and MMP13 are the major characterization of hypertrophic chondrocytes [141]. Meanwhile, the expressions of hyaline cartilage markers such as aggrecan, collagen type II, and SOX9 are decreased [141]. Although there is lacking attempt by using nanoparticles for preventing hypertrophy, these markers provide references for the selection of gene or drug delivery targets. Current evidence indicates that *NK3 homeobox 2* (*Nkx3.2*), *mothers against decapentaplegic homolog (Drosophila) 6* (*SMAD6*), *HDAC4*, *Chondromodulin 1*/*soluble Flt-1* and *ETS-related gene* (*ERG*) /*C-1-1*are also potential gene delivery targets to prevent chondrogenic hypertrophy [142]. Moreover, other developed therapeutic agents, including PTHrP, TGF-β, BMP-4/7/13, Dorsomorphin, MMP13 inhibitor, Erk1/2 inhibitor, Rac1 inhibitor and FK506, can be employed in combination with nanoparticles for delivery [142]. More importantly, by the adding above discussed bioactive ECM compositions (e.g., collagen), the efficacy of preventing hypertrophy can be further improved [125].

#### Cartilage Maintenance by Improving Chondrocytes Survival

Chondrocyte survival is important for the maintenance of cartilage matrix. Nanoparticles have been utilized to inhibit apoptosis (Fig. 7c). For example, berberine-loaded chitosan nanoparticles can effectively improve cell survival and ameliorate OA [82]. In fact, anti-inflammation, antioxidant stress, and the prevention of chondrogenic hypertrophy are all beneficial for cell survival. Adequate autophagy regulation is another strategy to promote chondrocyte survival. Normal chondrocytes express high levels of autophagy promoted by adenosine monophosphate-activated protein kinase (AMPK) and Sirtuin 1 to remove damaged and dysfunctional organelles and proteins [143, 144]. In OA, reduced autophagy in chondrocytes leads to increased catabolic processes and cell death [6, 143, 144]. Some nanoparticles have been fabricated to activate autophagy to mediate ROS scavenging, which is beneficial for relieving OA symptom [145]. Yan et al. fabricated the cationic amphipathic peptide-based nanoparticles for siRNA delivery in a mouse OA model [146]. By suppressing NF-κB, chondrocyte autophagic activity can be promoted through inhibiting mammalian target of rapamycin (mTOR), demonstrating its ability to permeate the dense cartilage matrix and treat OA [146].

### Symptomatic Treatment

#### Pain Relief by Inhibiting Inflammation

Pain is the predominantly symptoms of OA, caused by the activation of nociceptive pathways [147]. Inflamed tissues in OA express proalgesic molecules such as nerve growth factor (NGF), bradykinin receptors and tachykinin which is responsive for pain activation [147]. Therefore, pain management for allaying OA symptoms is dominated by the inhibition of articular inflammation. Nanoparticles can enhance the therapy efficiency of anti-inflammatory drugs, such as steroid and nonsteroidal anti-inflammatory drug (NSAID) (Fig. 7d) [140, 148]. IL-1β, TNF, and IL-6 modulate disturbed metabolism and enhanced catabolism in OA joint [149]. Nanoparticles loaded with selective inhibitors block these proinflammatory cytokines’ production and counteract the degradation of cartilage in OA [122, 150–152]. Currently used selective inhibitors include diacerein (against IL-1), interleukin-1 receptor antagonist (against IL-1), tocilizumab (against IL-6), and chloroquine (against TNF-α) [122, 150–152]. Besides that, cell-penetrating anti-inflammatory peptide KAFAKLAARLYRKALARQLGVAA (KAFAK) with the ability to suppress IL-1, TNF-α, and IL-6 has also been used in combination with nanoparticles [153–155].

Nanoparticles-based gene delivery to interrupt unnecessary gene expression in specific target cells, such as macrophage and chondrocyte, is also promising to inhibit joint inflammation. siRNA-loaded nanoparticles directly inhibit the expression of inflammatory factors such as *IL-1β*, *TNF*, and *IL-6* [63]. IL-1β and TNF-α can promote the expression of cyclooxygenase-2 (COX-2) and inducible nitric oxide (iNOS) synthase, leading to the production of prostaglandin E2 (PGE2) and nitric oxide (NO). siRNA delivery to silence the expression of inflammation-related genes such as *COX-2*, *iNOS*, and *MMP-9* is another strategy to suppress inflammation-associated catabolism [64, 156]. Besides, gene delivery targeting some signaling, such as NOTCH and NF-κB, is also possible to contribute to inflammation inhibition, resulting in retarded cartilage damage and bone erosion [146, 157].

#### Against Oxidative Damage

In OA, mitochondrial dysfunction leads to excessive production of reactive oxygen species (ROS) and downregulation of antioxidants such as superoxide dismutase, catalase, and glutathione peroxidase [6]. Excessive production of ROS increases apoptosis in chondrocytes by increasing mitochondrial DNA (mtDNA) damage, which further results in ECM degradation and joint inflammation [158]. Antioxidant supplements, mediators of various ROS pathways, and free radical scavengers are being investigated to protect cartilage against oxidative stress damage [159, 160]. Therapeutic agents-loaded nanoparticles have been designed to directly or indirectly protect cartilage against oxidative stress damage (Fig. 7e) [145, 161]. For example, manganese dioxide can catalyze the breakdown of hydrogen peroxide (H_2_O_2_), a key radical that is derived from O^2−^ to downregulate ROS level [161]. In a rat model, manganese dioxide nanoparticles with suitable physicochemical properties (less than 20 nm and cationic) can address issues of rapid release and achieve cartilage protection [161].

### In situ Cartilage Regeneration

With the progression of the OA and chondroptosis, a major challenge for attenuating the progression of cartilage degradation is the inability of the resident chondrocytes to lay down a new matrix [6, 162]. Cartilage does not regenerate due to the hostile local microenvironment and a limited supply of endogenous cells; as such, the rapid recruitment, migration, and infiltration of more joint-resident endogenous stem cells can provide better outcomes. Ideally, these recruited stem cells will be stimulated to differentiate into chondrocytes along with matrix synthesis to achieve in situ cartilage regeneration. In situ cartilage regeneration mainly contains two steps—recruitment of endogenous stem cells and chondrogenic differentiation (Fig. 7f, g).

#### Recruitment of Joint-resident Endogenous Stem Cells

Several types of MSCs including chondroprogenitor cells, synovium-derived MSCs, synovial fluid MSCs, bone marrow MSCs, fat pad-derived stem cells and MSCs in Ranvier groove are potential candidates for the recruitment into the defected cartilage [163]. The goal of recruitment is possible to be achieved by using nanoparticles decorated with advantageous cytokines or other active molecules (Fig. 7f). Cytokines including chemokines (e.g., CXCL8, CXCL12, CCL2, CCL3, CCL20) and grow factors (e.g., TGFs, IGF, FGFs, PDGF, BMPs) have been reported to facilitate MSCs homing and migration within cartilage [163]. Nanoparticles can be targeted to defect areas following active or passive delivery. With the degradation of nanoparticles and the release of cytokines, endogenous progenitor and stem cells can be recruited to synthesize and deposit nascent proteins and remodel the local microenvironments. Studies in future should fully consider the unfavorable effects of chemokines including activated inflammation, aggravated ECM catabolism, impeded chondrogenic differentiation, induced apoptosis and developed pain symptoms [163]. Moreover, it is also important to consider both the pathological mechanisms of OA and heterogeneity of stem cells. Biophysical and biochemical characteristics of nanoparticles should be designed to recruit specific subset of stem cells according to the pathological features.

#### Promoting Chondrogenesis

TGF-β family plays a critical role in skeletogenesis and OA progression. TGF-β2 or TGF-β3 deficient mice displayed skeletogenesis defects in the forelimbs, hindlimbs, and craniofacial bones [164]. In a chondrocyte-specific *Tgfbr2* knockout mice, higher expression of *Runx2*, *Mmp13*, *Adamts5*, and *Col10* along with increased hypertrophic chondrocyte numbers, early osteophyte formation, and increased subchondral bone mass are found, resembling the process of OA development [165, 166]. TGF family has been the most popular and widely investigated grow factor for cartilage repair. Intra-articular delivery TGF-β-loaded nanoparticles have been proved to stimulate ECM production, downregulate matrix-degradation, form hyaline cartilage, and therefore attenuate OA progression [70, 167, 168].

IGF-1 decreases in spontaneous OA, which aggravates articular cartilage lesions [169, 170]. IGF-1-loaded nanoparticles have also been successfully used to enhance cartilage repair and promisingly inhibit the progress of OA [40, 171]. IGF-1 promotes chondrogenesis of mesenchymal stem cell (MSC) and mediate chondrocyte phenotype [172, 173]. In addition, IGF-1 not only enhances the synthesis of proteoglycans and collagen type II, but also inhibits the ECM degradation by decreasing the production of matrix metallopeptidase [174, 175].

FGF-18 is a well-known anabolic growth factor which is involved in chondrogenesis and beneficial for cartilage repair [176]. Upregulation of FGF-18 induces the formation of cartilage with increased synthesis of matrix, and the in vivo delivery relieves the symptom of OA and promotes cartilage repair [176, 177]. *FGF-2* deletion induces accelerated spontaneous and surgically induced OA which can be reversed by subcutaneous administration of recombinant FGF-2 [178]. However, controversial roles have been reported that FGF-2 exerts catabolic effects that displays by the upregulated matrix-degrading enzyme production and down regulated ECM accumulation [179–181]. Therefore, the pharmacological actions and mechanisms should be fully confirmed before the therapy application. The BMPs as a family of growth factors have been widely applied for bone regeneration, while the promotion of chondrogenesis is another property [182, 183]. Both Smad-dependent and Smad-independent BMP pathways are required for chondrogenesis, and Indian hedgehog (IHH)/parathyroid hormone-related protein (PTHrP) and FGF pathways are key downstream targets [184]. It is possible to apply BMPs to improve both cartilage and subchondral bones functions. Of note, to date, there is lacking attempt by using nanoparticles to delivery FGFs and BMPs for either preventing or treating OA. Further preclinical experiments are required to investigate the feasibility and efficacy of these novel approaches.

Kartogenin (KGN) as a small bioactive molecule to promote chondrogenic differentiation of stem cells was first reported by Johnson et al. in 2012 [185]. KGN-loaded nanoparticles have been shown to play a critical role in chondrogenesis and promote cartilage repair in vivo [131, 132, 186].

Nanoparticles-based gene delivery has been explored for promoting chondrogenesis (Fig. 7g). Nanoparticles delivered plasmid encoding *TGF-β1* can increase *TGF-β1* expression in chondrocytes and therefore promote the proliferation [84]. PLGA nanoparticles encoding *TGF-β1* can be transfected to seed cells such as adipose-derived stem cells to upregulate expression of chondrogenesis-related genes such as *COL2a1*, *SOX9*, and *ACAN* [133]. SOX9 is an essential transcription factor for the chondrogenic differentiation which is crucial for *COL II* and *ACAN* expression [187]. *SOX9* genes in combination with PLGA nanoparticles increase the transfection efficiency into human mesenchymal stem cells for chondrogenesis [71].

### Perspective of Novel Therapeutic Schemes

#### Targeting Synovial Membrane and Subchondral Bone

**Fig. 8:**
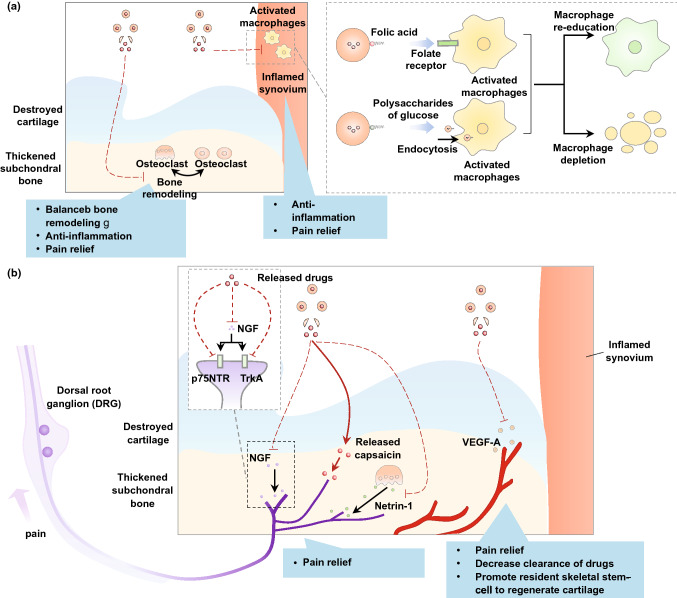
Other potential target tissues in addition to cartilage according to the known pathological mechanisms. **a** Schematic graph illustrates nanoparticles-based therapy targeting synovial membrane and subchondral bone. **b** Schematic graph illustrates nanoparticles-based therapy targeting nerves and blood vessels. Abbreviations: VEGF-A, vascular endothelial growth factor A; NGF, nerve growth factor; TrkA, tropomyosin-receptor-kinase A; p75NTRs, p75 neurotrophin receptors

Synovial membrane inflammation increases production of the proteolytic enzymes and ROS, which aggravates matrix degradation and contributes to OA progression [6]; therefore, it has been recognized as a diagnosis and/or therapy target [188]. Macrophage-associated inflammatory infiltrate can be found in OA cartilage, and the ablation of macrophages is beneficial for cartilage health and joint integrity [189]. Active targeting can be used for labeling activated macrophages as the diagnosis of OA cartilage (Fig. 8a). For example, folate receptor (FR) is expressed by activated macrophages in the inflammatory environment. Nanoparticles conjugated with near-infrared dye and folic acid (FA) can be used as probes to detect activated macrophages, quantify severity and deliver drugs [190, 191]. Another targeting strategy is using nanoparticles with the conjugation of certain polysaccharides of glucose such as dextran which can be selectively internalized by macrophage cells due to their expression of dextran-binding C-type lectins and scavenger receptors [192]. The major principles for the nanoparticles-based therapy targeting synovial membrane include macrophage depletion or re-education [193]. PEGylated Ag nanoparticles decorated with folic acid (FA) can target inflamed synovial membrane to induce M1 macrophage apoptosis and M2 macrophage polarization [191]. In addition, it is also possible to decrease macrophage recruitment by inhibiting of C–C Motif Chemokine Ligand 2 (CCL2)–C–C chemokine receptor type 2 (CCR2) signaling pathway or inhibit macrophage survival by inhibiting colony-stimulating factor 1 receptor (CSF1R) signaling [194]. Notably, cell membrane-camouflaged nanoparticles, prepared by the fusion of cellar membrane with nanoparticles, exhibit great potential in elongating circulation time, evading immune responses, and effective targeting on specific tissues or cells [195]. For example, macrophage-derived microvesicle-coated nanoparticles, which mimic macrophages, can targeting Macrophage-1(Mac-1) and CD44 to contribute to the drug delivery [196]. In addition, coating nanoparticles with neutrophil membrane are an ideal decoy of neutrophil-targeted biological molecules. These nanoparticles neutralize proinflammatory factors and inhibit synovial inflammation [197].

Dysregulated subchondral bone remodeling in OA leads to bone attrition and sclerosis, subchondral plate thickens, and osteopenic subchondral cancellous bone [198–201]. Hence, drug delivery to recover subchondral bone function is also needed (Fig. 8a). However, there is still a lack of acknowledged treatment based on the regulation of subchondral bone function. Before intervention for subchondral bone reconstruction disorders, much work needs to be done to clarify the pathogenesis of subchondral bone disorders, the regulatory relationship with cartilage, effective targeted drugs and the time window for intervention.

#### Pain Relief Targeting Nervous System

Inflammatory pain in OA is controlled by both immune system and nociceptive neurons [147]. Inflammatory pain signals produced by pro-inflammatory cytokines and chemokines stimulations (e.g., TNF, IL-1, granulocyte–macrophage colony-stimulating factor (GM-CSF), NGF and prostaglandin E2 (PGE2)) can be detected by somatosensory neurons (nociceptors) [147]. As the cell bodies of somatosensory neurons are clustered in dorsal root ganglia (DRG), signals are firstly carried to the dorsal horn of the spinal cord via dorsal root ganglia then transmitted to the brain via central axonal terminals [147]. Multiple tissues surrounding cartilage (including the synovium, ligament, osteochondral junction, and meniscus) are densely innervated [202–205], which may be the new therapy targets for pain relief (Fig. 8b).

Neurotrophins includes NGF, brain-derived neurotrophic factor (BDNF), neurotrophin 3 (NT3) and neurotrophin 4 (NT4) are important in afferent sensitization of nociceptors in OA [147, 206–208]. Since NGF is highly expressed in the inflamed synovium and at the osteochondral junction for afferent sensitization of nociceptors, underlying pain management strategy is to inhibit NGF signal transduction by using the related inhibitor, NGF-neutralizing monoclonal antibodies and/or siRNA encapsulated nanoparticles [207, 208]. The blocking of NGF receptor including tropomyosin-receptor-kinase A (TrkA) and p75 neurotrophin receptors (p75NTRs) inhibits nociceptor sensitization by NGF [209]. In addition, capsaicin derived from chili peppers induces excitation and subsequent desensitization, which is also a delivered candidate for pain relive [210].

Sensory innervation in OA can be mediated by osteoclast-initiated subchondral bone remodeling [202]. Netrin-1 secreted by osteoclasts induce sensory innervation and OA pain through its receptor Deleted in Colorectal Cancer (DCC) [202]. Possible pain relief strategy including osteoclast inhibition (e.g., gene delivery by nanoparticles for inhibition RANKL production) or netrin-1 production inhibition (e.g., gene delivery by nanoparticles for inhibition netrin-1) is emerging.

#### Pain Relief Targeting Blood Vessel

Angiogenesis is another factor that contribute to structural damage and OA pain [211, 212]. Increased vascularization in OA is mainly caused by inflammation-associated macrophage infiltration and reduced cartilage resistance to angiogenesis [211]. As some pathways and molecules stimulate both vascular cells and nerve growth, sensory nerves often grow along new blood vessels which disrupt the osteochondral junction and penetrate non-calcified articular cartilage [211, 212]. Consequently, both vascularization and inflammation contribute to nerves sensitization and increased pain [211, 212]. In addition, angiogenesis in OA cartilage increased clearance of drugs. Moreover, anti-angiogenesis is capable of promoting resident skeletal stem-cell to regenerate cartilage [213]. Therefore, antiangiogenic nanoparticles to relieve pain and slow the progression of joint damage is a potential strategy for OA therapy (Fig. 8b).

Anti-angiogenesis can be divided into direct inhibition (e.g., targeting vascular cells) or indirect inhibition (e.g., reducing inflammation, inhibiting the matrix degradation and osteochondral channel formation). Nanoparticles for anti-angiogenesis in OA has not been reported previously. Anti-angiogenesis nanoparticles in tumor therapy also have potential for the treatment of OA since the neovascularization mechanisms are similar. Several growth factors (e.g., vascular endothelial growth factor A (VEGF-A), bFGF and TNF-α) have been proved to stimulate quiescent vascular endothelium to enter the cell cycle [214], which are possible to be the main targets for nanoparticles anti-angiogenesis therapy (Fig. 5b). For example, Au nanoparticles bind to heparin-binding growth factors (HB-GFs) such as VEGF165 and bFGF to inhibit angiogenesis [215]. Ag nanoparticles show anti-angiogenic activity by downregulating PI3 K/Akt pathway and inhibiting HIF-1α protein accumulation to further inhibit the expression of VEGF-A [216, 217]. Nanoparticles-based gene delivery to inhibit expression of genes such as VEGF and HIF-1α is also promising for anti-angiogenesis therapy [218].

### Personalized Therapy According to the Pathology Stages

The clinical heterogeneity of OA affects the therapeutic outcome, as different phenotypes need specific therapeutics. Therefore, the design of “smart” nanoparticles with diverse physicochemical properties for personalized therapies needs to be based on different etiological factor and pathological mechanisms. For example, in senile OA, disease progression is associated with increased chronic inflammation and mitochondrial dysfunction [219]. In contrast, in post-traumatic OA, a vigorous inflammatory response occurs very early after joint injury and then sustained at a lower level [220]. The determination of the optimal approach and timing of anti-inflammatory interventions will provide reference for the control release of nanoparticles. Meanwhile, manipulating one aspect of mechanism may affect another. For example, inhibiting inflammation at optimal stage may also indirectly promote chondrogenesis [221]. At the later stage of OA with severe cartilage defects, anti-inflammatory therapy may only relieve the symptoms. Modifications of nanoparticles to enhance function of cartilage protection and/or promotion of chondrogenesis are needed.

The convergence of biomaterial science and biomedicine opens unprecedented opportunities for the diverse medical applications of nanoparticles. Thus, in the future, with the development of noninvasive diagnostic technology, it is worthwhile to identify the pathological characteristics of each patient's joint before modification of size, charge, and surface-functional properties of nanoparticles for individualized treatment.

## Perspective of “Smart” Bioresponsive and Multi-modality Nanoparticles

### Bioresponsive Nanoparticles for Controlled Delivery

Tradition drug delivery system provides sustainable drug release; however, it is likely to cause sub- or supra-therapeutic drug levels locally since the disease activity changes over time. Ideal “smart” nanoparticles ensure that the drug will be released with proper rates at the target sites, and therefore minimize non-specific toxicity and enhance the therapy efficacy. The controlled release, depending on the biological signals (e.g., pain intensity) or pathological abnormalities (e.g., severity degree) of OA, can be achieved by responding to endogenous and/ or exogenous stimulus. The release of external-responsive nanoparticles can be subjectively controlled (e.g., when pain occurs) by a physician or patient, whereas the release of internal stimuli-responsive nanoparticles can be triggered depending on objective pathological changes.

Making full use of the nanomechanical properties is beneficial to regulate the smart intra-articular drug delivery. The basal drug release rate and trigger energy should be fully balanced during the design and fabrication. Nano-motors as a research hotspot have been regarded as the new generation of drug delivery system owing to the tiny size and unique mobility [222]. External-responsive nanoparticles, precisely powered by external field, such as magnetic field, electric field, and ultrasonic field, can be developed as nanomotors to penetrate cartilage tissue and trigger the drug release [222, 223]. Joint movement directly affects the retention and penetration of nanoparticles, and at the same time, the heat generated by friction also indirectly affects the degradation of nanoparticles. The design of mechanical stimuli or thermal responsive nanoparticles could be inspired by the joint movement. This section highlights the development opportunities of stimulus-responsive nanoparticles for inter-articular drug delivery.

#### External-Responsive Nanoparticles

**Fig. 9:**
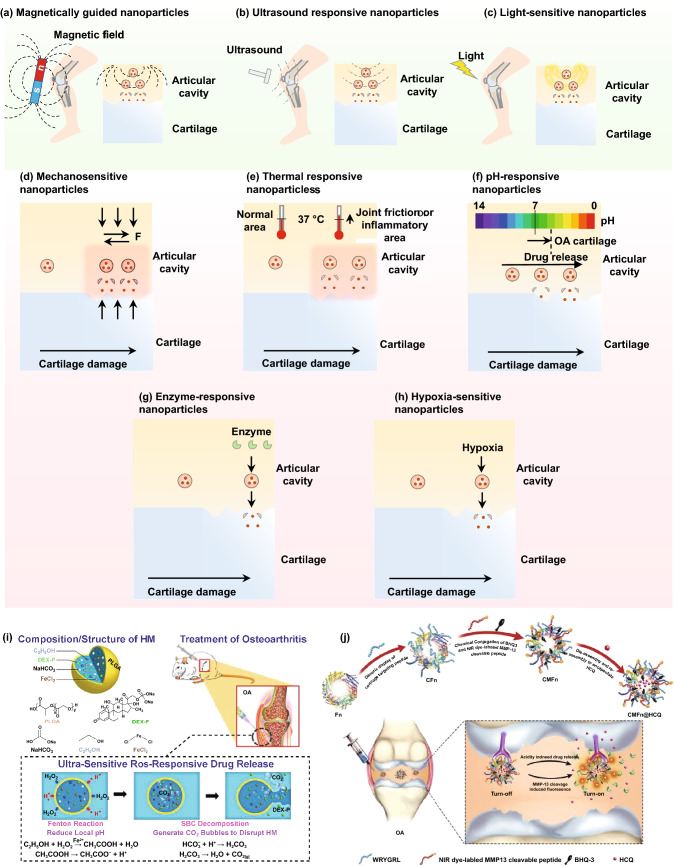
Potential therapeutic strategies by using stimuli-responsive nanoparticles for control delivery in OA. **a**–**c** Schematic graphs illustrate external-responsive nanoparticles for OA therapy. **d**–**h** Schematic graphs illustrate internal stimuli-responsive nanoparticles for OA therapy. Reproduced with permission [242]. Copyright © 2015 American Chemical Society. **i** Example of pH responsive nanoparticles for OA imaging and therapy. **j** Example of enzyme responsive nanoparticles for OA therapy. Reproduced with permission [53]. Copyright © 2019 Elsevier Ltd. Abbreviations: OA, osteoarthritis Copyright © 2015 American Chemical Society. **i** Example of pH responsive nanoparticles for OA imaging and therapy. **j** Example of enzyme responsive nanoparticles for OA therapy. Reproduced with permission [53]. Copyright © 2019 Elsevier Ltd. Abbreviations: OA, osteoarthritis

Magnetically guided nanoparticles including iron oxide nanoparticles, iron oxide hybrid nanoparticles, and other magnetic nanoparticles may be applied for the controlled delivery by a magnetic guidance under a permanent magnetic field or alternating magnetic field [223, 224]. An extracorporeal magnetic field near cartilage can be applied for magnetic guidance and on-demand release after the intra-articular injection of a magnetically responsive nanoparticles (Fig. 9a). For instance, Jafari et al. utilized dynamic magnetic fields to guide the transport of magnetic nanoparticles through the entire thickness of bovine articular cartilage [225]. The alternating magnetic field induces a nearly 50 times increase in magnetic nanoparticles transport as compared with static field conditions [225]. Moreover, magnetic nanoparticles can be used as cell labels and guided seeding for cartilage repair [226]. Meanwhile, the magnetic nanoparticles (e.g., Fe_3_O_4_)-mediated physical stimuli promote chondrogenic differentiation in vitro by enhance level of sulfated glycosaminoglycan (sGAG) and collagen synthesis [227]. It should be noted that whether the increased temperature under the alternating magnetic field has adverse effect on the cartilage repair is unclear and should be confirmed before the therapy application. Ultrasound waves can trigger controlled release of drugs through the thermal and/or mechanical effects (Fig. 9b) [224]. Cartilage penetration depth can be regulated through cavitation phenomena or radiation forces [224]. Since pulsed ultrasound has been proved to be effective in pain relief in OA, therapy based on ultrasound responsive nanoparticles may lead to both controlled drug release and function improvement [228, 229]. Photosensitiveness-induced structural modifications of the nanoparticles can trigger drug release in response to light (Fig. 9c) [224]. The irradiation wavelength and power can be adjusted to achieve noninvasive controlled release by cleaving the light-sensitive chemical bonds or assembling in response to light [230–232]. Photoresponsive nanoparticles, which achieve drug release in response to ultraviolet, visible or near-infrared (NIR) regions, can be engineered. However, due to the strong scattering properties of soft tissues, the penetration depth of visible light and ultraviolet light is limited to less than 10 mm, so they are not suitable for intra-articular delivery [224]. NIR laser (700–1,000 nm range) can replace the ultraviolet–visible light with a deeper tissue penetration (2–5 cm) [224]. For example, doxorubicin-loaded hollow gold nanoparticles convert the photon energy adsorbed during irradiation (808 nm) into heat to trigger drug release [233].

#### Internal Stimuli-Responsive Nanoparticles

OA (especially in knee), as a joint failure with myriad causes, is affected more by mechanical stress rather than systemic factor such as inflammation, aberrant metabolic regulation, and obesity [234]. The fabrication of mechanical stimuli-responsive nanoparticles has been reported previously [235, 236]. For example, Holme et al. fabricated lenticular liposomes made from an artificial 1,3-diaminophospholipid which are stable under static conditions but release their encapsulated contents at elevated shear stress [235]. It is theoretically possible to use such nanoparticles on the wear and tear surface to achieve automatic release of drugs for improved treatment outcome (Fig. 9d). The general concept is based on the assembly and disassembly of mechano-responsive structures under the changing mechanical environment.

Thermal responsive hollow nanoparticles have been reported to enhance the delivery efficiency in OA (Fig. 9e) [155, 237]. Further functional improvements of thermal responsive nanoparticles can be learned from experience in cancer research [238]. Generally, thermal responsive nanoparticles in cancer therapy remain stable at body temperature (∼37 °C) and release drug rapidly in a higher temperature (∼40–42 °C) [223, 224]. Potential of using such nanoparticles in OA therapy is based on the two possible reasons. Firstly, the joint temperatures in OA (especially in the defected area) may be higher which is related to greater joint friction effects and retarded cooling [239]. Secondly, joint inflammation may also lead to high local temperature.

Lower pH during cartilage degeneration alters the proteoglycans structure and concentration, leading to decreased viscosity [240]. pH‐responsive nanoparticles may tune drug release and cell uptake in OA cartilage for achieving high therapeutic performance (Fig. 9f). For instance, chitosan as a pH responsive polysaccharide has been utilized to fabricate pH‐responsive nanoparticles which can release faster in acid environment (pH ~ 5.5) than neutral environment [241]. In a sodium bicarbonate-encapsulated particle, the release of anti-inflammatory drug can also be triggered by an acidic milieu induced decomposition reaction (Fig. 9i) [242]. Lysosomal molecules are upregulated in OA which involved in the modulation of chondrocytes death [243]. The acidic luminal pH in OA lysosome compartment is also likely to trigger the release of pH sensitive particles in defected cell for effective intracellular drug accumulation. The feasibility still needs to be verified as pH-sensitive nanoparticles may lead to leakage of lysosomal enzymes by disturbing lysosomal membranes and further cause autophagy and cell death.

Since matrix-degrading enzymes, such as MMP3, MMP13, and ADAMTS5, are upregulated in OA cartilage, they can be exploited to achieve enzyme-mediated drug release (Fig. 9g). For example, nanoparticles containing matrix metalloproteinase substrate peptide (e.g., H2N–GPLGVRGC–SH as MMP-13 cleavable specific peptide substrate) undergo morphological transition for drug release when react with matrix metalloproteinase in a mouse OA model (Fig. 9j) [53].

As an avascular tissue, cartilage is hypoxic in nature. This property can be utilized to design hypoxia-sensitive nanoparticles for the controlled release of drug in cartilage. Hypoxia-sensitive nanoparticles are normally constructed with hypoxia-sensitive materials or derivatives such as 2-nitroimidazole, nitroimidazole, metronidazole, azobenzene, nitro-benzene derivatives and iridium (III) complexes, etc. [223]. Before the design, oxygen tension in health cartilage and OA cartilage needs to be confirm.

### Multi-modality Nanoparticles

#### Nanoparticles in OA Diagnosis

**Fig. 10:**
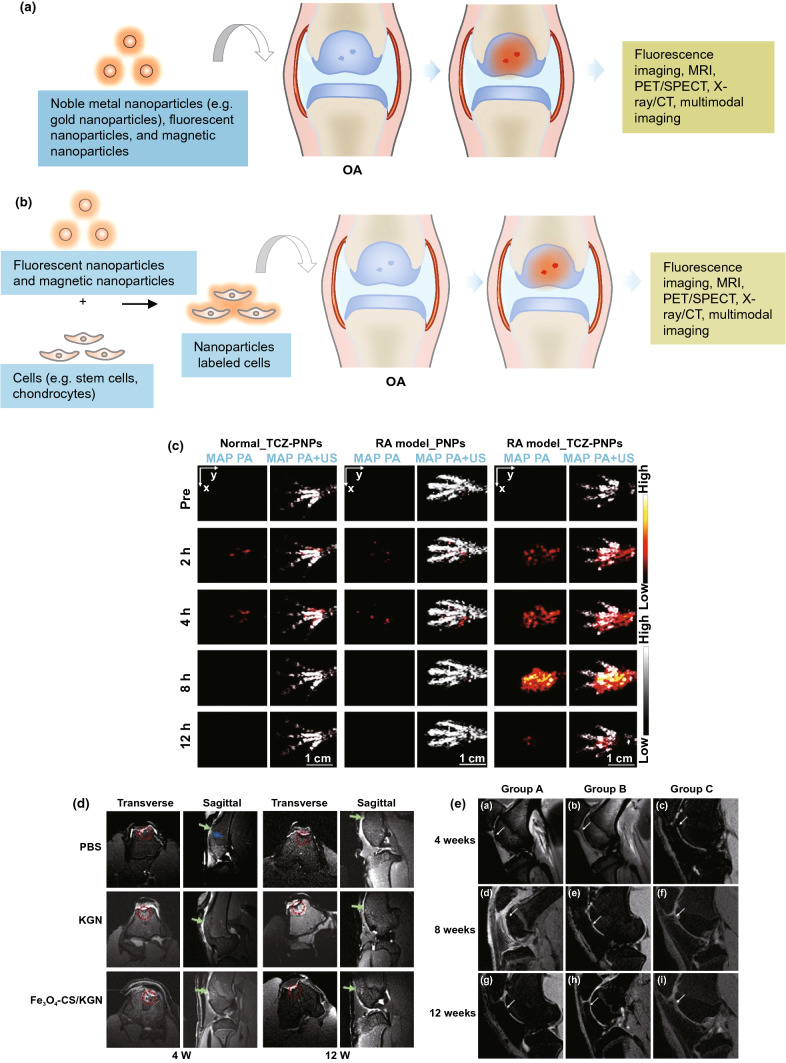
Multiples applications of nanoparticles in OA. **a** Schematic graph illustrates application of nanoparticles in OA diagnosis. **b** Schematic graphs illustrate application of nanoparticles in cell tracking.** c** Example of fluorescent labeled nanoparticles in cartilage diseases. Reproduced with permission [252]. Copyright © 2020 Wiley‐VCH GmbH. **d** Example of magnetic nanoparticles in OA diagnosis. Reproduced with permission [186]. Copyright 2020, Ivyspring International Publisher. **e** Example of magnetic nanoparticles in cell tracking. Reproduced with permission [260]. Copyright 2012, Springer Nature. Abbreviations: CT, computed tomography; MRI, magnetic resonance imaging; OA, osteoarthritis; PET, positron emission tomography; SPECT, single-photon emission computed tomography

Cartilage degradations in OA can only be diagnosed when the size of defected area is larger enough. Consequently, prompt diagnosis is often important for early treatment, however difficult due to the lack of diagnostic options. To fill this gap, there is a significant growth of interest in the use of nanoparticles as optical imaging probes in diagnostics (Fig. 10a, c, d). Deeper imaging penetration, photostability, and biocompatibility should be fully considered.

Noble metal nanoparticles (e.g., gold nanoparticles), fluorescent nanoparticles, and magnetic nanoparticles, with advantages of increased the sensitivity, better detection capabilities and ease of operation have been studied for possible application of OA diagnosis [54, 186, 244–248]. The advantages of fluorescent dyes include high selectivity, high sensitivity, and high fluorescence quantum yield; however, obstacles including potential toxicity, poor fluorescence stability limit their medical use. Metal oxide nanoparticles can be used as contrast agents in magnetic resonance imaging. For diagnosis, metal nanoparticles are more stable than organic fluorescent labels and the surface can be modified (e.g., by polymers and surfactants). Gd and Mn elements are used as a T1-weighted contrast agent for magnetic resonance (MR) imaging [249, 250]. However, drawbacks such as toxicity and tissue accumulation limit the applications [251]. Fe element-based nanoparticles with superparamagnetism have been studied as nanoprobe to provide dark T2-weighted imaging [186, 244–246]. For example, synthesized magnetite Fe_3_O_4_ nanoparticles penetrate cartilage and the size of impregnated particles shows the degree of cartilage degradation [245].

Gold nanoparticles-based probes are appealing for the diagnosis of OA. They are inert and therefore are biocompatible and non-toxic. The surfaces of gold nanoparticles can be easily modified. Moreover, gold nanoparticles can be incorporated into larger structures such as polymers or liposomes for enhanced diagnostic applications [247]. For example, by conjugating gold nanoparticles with a FITC-modified ADAMTS-4- specific peptide (DVQEFRGVTAVIR), the gold nanoparticles probe has been developed for detection of aggrecanase [248]. When the probe is incubated with aggrecanase ADAMTS-4, the fluorescence intensity significantly increases [248]. As a result, the increased aggrecanase activity can be detected for early diagnosis of cartilage-degradation [248].

NIR‐II photoacoustic molecular imaging is emerging as a strategy for effective diagnosis. Compared to conventional optical imaging modalities, NIR-II photoacoustic molecular imaging is generated by the absorption of light by a molecule under short pulse laser irradiation, which overcomes the common optical diffusion limit [252–254]. NIR‐II-conjugated polymer nanoprobe displays effective noninvasive diagnosis of cartilage disease [252].

Nanoparticles probes can also be designed based on the changes of inflammation states during OA. As the macrophage activity often increases in OA, a nanoprobe has been fabricated by conjugating hyaluronic acid with near-infrared dye and folic acid (FA), to bind to folate receptors of macrophages [190]. In addition, the overproduction of nitric oxide (NO) in OA stimulates the production of proinflammatory cytokines, such as TNF α and IL-1β [255]. By encapsulating the NO sensing molecules (e.g., 4-amino-5-methylamino-2′,7′-difluorofluorescein Diaminofluorescein-FM (DAF-FM)) within nanoparticles, NO release can be monitored which permits the indirect real-time evaluation of OA development [255].

#### Nanoparticles in Cell Tracking

Advances in cell therapy have emphasized the role of chondrocytes and mesenchymal stem cells (MSCs) with chondrogenic differentiation ability, migratory, anti-inflammatory and/or immunosuppressive properties in treating cartilage lesions (Fig. 10b, e) [256]. A noninvasive means of imaging and tracking the cell fate after in vivo implantation could be extremely valuable. Quantum dots such as fluorescence-labeled mesoporous silica nanoparticles and superparamagnetic iron oxide (SPIO) nanoparticles have been developed for in vitro and in vivo bioimaging [257, 258]. The basic principle for the cell-labeling agents is that enough number of nanoparticles should bind to cells to be detectable. Besides, nanoparticles should not interfere the cellular functions such as cell viability and differentiation capacity. By using superparamagnetic iron oxide nanoparticles (SPIONs) cell labeling, magnetic resonance imaging (MRI) can visually monitor the in vivo dynamic biodistribution of implanted cell. With good biocompatibility, SPIONs do not affect cell viability, proliferation, and differentiation capabilities [259, 260].

#### Theranostic Applications of Metal Nanoparticles

Theranostics are combinatorial approaches that aim to deliver therapy and examine the effect at the same time. Combining the nanoparticle-based drug delivery strategy with the diagnostic techniques of nanoparticles provides the possibilities to achieve this aim [261]. For example, chitosan-modified Fe_3_O_4_ nanoparticles have been fabricated for delivery KGN, which can be used as for both diagnosis under magnetic resonance (MR) imaging and osteochondral regeneration [186]. Noble metal nanoparticles, such as gold, silver, or a combination of both, with advantages of high surface-to-volume ratio, ease of synthesis and highly tunable optical properties, can comprise nucleic acids (DNA/RNA), drugs and biocompatible polymers (e.g., polyethylene glycol and PEG) for delivery. In addition, there unique properties can be utilized (e.g., gold nanoparticles for diagnostics [262]; silver and platinum (Pt) nanoparticles as reactive oxygen species scavengers [263]). For example, silver nanoparticles have been explored to alleviating joint inflammation via reeducate macrophages [191]. It is possible to expect more effective therapy through the diagnosis and prediction of pathological process to control of drug release by adding stimuli-responsive components in nanoparticles. However, the major shortcoming is that noble metal NPs are difficult to be degraded from organisms which may interfere medical diagnoses. Moreover, the degradation by lysosomes within cells may produce toxic free metal ions such as Ag^+^ and Au^+/3+^, which affect cell homeostasis [264]. As such, it is important to balance action, clearance and toxicity. Zinc (Zn), and magnesium (Mg), as the best-explored biodegradable metals for orthopedic applications, hold promise to be fabricated as the moiety of nanoparticles to solve the non-degradable problems [265, 266]. After degradation, the cations (e.g., Zn ^2+^, Mg^2+^) may be resorbed by the negative charged cartilage matrix. However, Zn^2+^ in OA increases the synthesis and secretion of matrix-degrading enzymes which aggravated the progression of OA [267]. In contrast, Mg nanoparticles may be more promising for OA therapy as intra-articular injections of Mg^2+^ at 0.5 mol L^−1^ attenuate the progression of OA by inhibiting inflammation and matrix-degrading enzymes [268]. Moreover, particles containing magnesium powder can continuously evolve gaseous H_2_ which can also effectively mitigate joint inflammation [269]. Future smart design of theranostic nanomaterials needs to improve the stability of biomaterials and reduce toxicity for co-delivery of multiple components and more sensitive detection [270]. At the same time, how to design the bio‐inspired materials for better interaction with the pathological features needs to be explored [271, 272].

## Translation from ‘Bench to Bedside’

Dozens of nanoparticles including liposome, polymer, micelle, inorganic, nanocrystal and protein nanoparticles have received FDA approval and are currently available for clinical use for the therapy of disease such as kidney disease, cancer, sclerosis, and bone defect [273, 274]. Preclinical studies discussed above show the potential of nanoparticles to improve the drug delivery efficiency for the treatment of OA. However, to date, there is still limited clinical application for the therapy of OA. For example, FX006 as a PLGA-based drug delivery system for the treatment of OA in clinical trials results in clinically minimal improvement in pain relief [275]. To bridge this gap, there is a need to understand the challenges. Firstly, the basic principle for the clinical application is low toxicity. Toxicological studies on the application of nanoparticles in cartilage are still few. Besides, more efforts are needed to confirm the pharmaceutical stability and the in vivo behavior, such as cellular and molecular interactions. It is apparently a great challenge to choose effective pharmacological agents, given that the clinical efficacy and mechanisms (e.g., hyaluronic acid, glucosamine, and chondroitin) for OA therapy are still uncertain. More importantly, the modification of nanomaterials in basic research makes their structures and components more complex and their functions more diverse, which need high development cost for the clinical translation. In contrast, it is more feasible to evaluate the clinical safety and efficacy of nanomaterials with simpler structures and components. However, maybe it is “Easier Said Than Done.” For the avascular cartilage tissue, it is more difficult to ensure the efficacy with simple materials. Extensive efforts are needed for controlling the complexity without decreasing therapeutic efficacy.

## Conclusions

Current nanoparticles-based intra-articular delivery represents a new frontier arisen from an urgent need to address the issues of low drug retention. Ideal nanoparticles with suitable size, charge, and modification are supposed to penetrate the cartilage matrix easily and provide sustainable drug or gene delivery upon demand, which is conducive to the relief of OA symptom and cartilage regeneration. A comprehensive understanding of nanoparticle transport and nano–cartilage interactions in vivo is vital for improving therapeutic efficacy, avoiding or minimizing the adverse effects. Further advances are in progress to bring forth more “smart” diagnosis and therapy with the constant improvement of material technology and continuous evolvement of the understanding of OA. We anticipate that with better and fundamental understanding on cartilage–nanoparticle crosstalk, multidisciplinary collaborations, we will definitively be able to advance nanomedicine toward early diagnosis and effective therapy for OA.
